# Effects of 3,3′,5-triiodothyronine supplementation during ex vivo lung perfusion on ischemia/reperfusion injury in rat lungs

**DOI:** 10.3389/fmed.2026.1924438

**Published:** 2026-09-10

**Authors:** Ilaria Righi, Caterina Lonati, Michele Battistin, Francesco Damarco, Margherita Cattaneo, Luigi Vivona, Claudia Bonetti, Marcello Pinti, Valentina Selleri, Laura Fugazzola, Irene Campi, Angela Lombardi, Alessandro Cherubini, Mario Nosotti, Lorenzo Rosso

**Affiliations:** 1 Thoracic Surgery and Lung Transplantation Unit, Fondazione IRCCS Ca’ Granda Ospedale Maggiore Policlinico, Milan, Italy; 2 Center for Preclinical Research, Fondazione IRCCS Ca' Granda Ospedale Maggiore Policlinico, Milan, Italy; 3 Renal Research Laboratory, Unit of Nephrology, Dialysis and Renal Transplantation, Fondazione IRCCS Ca' Granda Ospedale Maggiore Policlinico, Milan, Italy; 4 Department of Anaesthesia and Intensive Care, IRCCS MultiMedica San Giuseppe Hospital, Milan, Italy; 5 Emergency and Intensive Care Department, ASST Papa Giovanni XXIII, Bergamo, Italy; 6 Department of Life Sciences, Modena, Italy; 7 Department of Health Science, University of Milan, Milan, Italy; 8 Department of Endocrinology, ASST Santi Paolo e Carlo, Milan, Italy; 9 Department of Endocrine and Metabolic Diseases, IRCCS Istituto Auxologico Italiano, Milan, Italy; 10 Precision Medicine and Biological Resource Center, Fondazione IRCCS Ca' Granda Ospedale Maggiore Policlinico Milano, Milan, Italy; 11 Department of Pathophysiology and Transplantation, University of Milan, Milan, Italy

**Keywords:** ex vivo lung perfusion, ischemia/reperfusion injury, mitochondrial DNA, small animal model, thyroid hormone

## Abstract

**Background:**

Ischemia/reperfusion injury (IRI) still represents a major determinant of both early and late complications of lung transplantation. Increasing evidence indicates that ischemia-induced molecular damage and energetic impairment are established before ex vivo lung perfusion (EVLP) and continue to progress over perfusion. Thyroid hormones support mitochondrial metabolism and cellular energetics and show multiple protective effects in different models of lung injury. We investigated, for the first time, the potential effects of 3,3′,5-triiodothyronine (T3) supplementation during experimental EVLP.

**Methods:**

An established rat model of EVLP was used. After procurement, lungs were either immediately perfused for 3 h (*n* = 10) or exposed to 2 h of ischemia (1 h-warm + 1 h-cold), followed by 3 h of EVLP (*n* = 10). In each condition, T3 (EVLP + T3 and Ischemia + EVLP + T3, *n* = 5 each) or an equal volume of saline (EVLP and Ischemia + EVLP, *n* = 5 each) were directly added to the perfusion fluid. Lung hemodynamics and airway pressure were monitored throughout the procedure. Perfusate samples were collected at time intervals to assess gas pressures, electrolyte balance, biomarkers of cell damage, and mitochondrial DNA (mtDNA) release. Biopsies were taken at the end of EVLP for cellular energetics evaluation and selected gene expression profiling. Samples collected from rats in resting conditions were used as reference (Native, *n* = 5).

**Results:**

T3 supplementation was associated with reduced pulmonary artery pressure and vascular resistance in uninjured lungs, while ischemic lungs showed improved CO₂ clearance. However, at the molecular level, T3-treated lungs showed no significant advantage compared to the corresponding control groups. Indeed, T3 administration was associated with an increased mtDNA release into the perfusate and induction of pro-apoptotic signaling. Moreover, T3 failed to restore the ATP depletion induced by ischemia and did not improve cell energy charge. Gene expression analysis showed only modest transcriptional modulation of stress and inflammatory pathways, without evidence of a protective molecular signature.

**Conclusion:**

Despite promising results in several lung injury models, our evidence indicates that T3 administration during EVLP does not exert protective effects but could rather exacerbate mitochondrial stress in lungs exposed to ischemia. These results suggest caution when considering T3-based interventions for organ reconditioning during EVLP.

## Introduction

Ischemia/reperfusion (IR) remains a critical challenge in lung transplantation, significantly contributing to early and long-term complications, including primary graft dysfunction (PGD) (1) and chronic lung allograft dysfunction (CLAD) (2, 3). While ex vivo lung perfusion (EVLP) has dramatically improved functional assessment, the procedure does not allow a full recovery from the initial deleterious events triggered by IR (4). Indeed, experimental and clinical studies showed that, despite preserved lung function, IR-related injury is already established at the biomolecular level during ex vivo perfusion (5–11). Moreover, although EVLP is generally associated with similar outcomes to conventional preservation (12–15), a delayed recovery (16–19) and an incomplete functional improvement (20, 21) have been reported in ex vivo-perfused lungs. Collectively, these observations indicate that a certain degree of cell injury continues to evolve during EVLP, suggesting the need for protocol optimization (22–26) and for complementary therapies (27–30) aimed at counteracting the detrimental consequences of IR.

Among the various pathophysiological mechanisms involved in IR injury, energy exhaustion, mitochondrial impairment, and reactive oxygen species (ROS) generation have emerged as key drivers of tissue damage (31, 32). More specifically, during the ischemic phase, the impairment of oxidative phosphorylation establishes an altered intracellular environment that leads to adenosine triphosphate (ATP) depletion and promotes massive ROS generation upon reperfusion (31, 33–35). The generalized mitochondrial dysfunction triggers a complex chain of subcellular events, culminating in induction of sterile inflammation (36–38) and cell death activation (31, 36, 37, 39).

Thyroid hormone (TH) could represent promising therapeutic agents to promote mitochondrial recovery, based on their well-established ability to support cell metabolism (40). While low 3,3′,5-triiodothyronine (T3) plasma concentrations are associated with worse outcomes in many acute and chronic stress conditions (41–45), T3 supplementation was reported to exert multiple protective effects in several models of injury and inflammation, including lung fibrosis (46, 47), hyperoxic lung injury (41, 48), pulmonary edema (48, 49), and ventilator induced-injury (50, 51). In type II alveolar epithelial cells exposed to different stressful stimuli, T3 supplementation was associated with improved bioenergetics and reduced mitochondria-mediated apoptosis following stress exposure (41, 46). Of note, in pulmonary epithelial cells exposed to cold ischemia (CI) followed by warm reperfusion, the addition of THs during cold storage promoted mitochondrial function, reduced apoptosis, and inhibited inflammatory gene expression (52). Finally, T3 exerted significant cardioprotective effects in a preclinical model of heart normothermic perfusion (53).

Building on these findings, the present research investigated, for the first time, whether T3 administration during EVLP exerts protective effects in lungs exposed to warm ischemia (WI). Our study was based on an established model of ex situ perfusion of the rat lung (54), allowing continuous monitoring of pulmonary function. The main outcomes were evaluation of cellular and mitochondrial oxidative damage, measured by mitochondrial DNA (mtDNA) release, and assessment of lung cell energetic status.

Given its long-standing clinical use, T3 supplementation during EVLP represents a biologically relevant strategy to investigate whether metabolic stimulation can modulate mitochondrial function and cellular energetics in this setting.

## Methods

### Study design

The present research was designed according to the “Planning Research and Experimental Procedures on Animals: Recommendations for Excellence” (PREPARE) guidelines (55).

Animals were assigned to one of the following experimental groups (*n* = 5): (1) Native, lung biopsies were collected from rats in resting conditions; (2) EVLP, lungs were procured and immediately perfused for 3 h using Steen Solution™ (XVIVO, Mölndal, Sweden); (3) EVLP + T3, lungs were procured and immediately perfused for 3 h using Steen Solution™ supplemented with 1 μΜ Τ3; (4) Ischemia, lungs were exposed to 1 h of WI followed by 1 h CI; (5) Ischemia + EVLP, ischemic lungs were perfused for 3 h using Steen Solution™; (6) Ischemia + EVLP + T3, ischemic lungs were perfused for 3 h using Steen Solution™ supplemented with 1 μΜ Τ3.

Randomization and blinding approach adopted in the present study are described in the Supplementary Methods 1.1 and Supplementary Figure 1.

Treatment was performed using a commercially available formulation of synthetic T3 (Sigma-Aldrich, Merck KGaA, Darmstadt, Germany), which, after appropriate dilution (Supplementary Methods 1.2), was added to the perfusion fluid at 20 min of reperfusion, to achieve a final concentration of 1 μM. An equal volume of saline was added to the untreated groups (EVLP and Ischemia + EVLP).

### Animals and sample size calculation

Experiments were conducted under authorization from the Italian Ministry of Health (approval number 1108/2020, issued on 13/11/2020).

The study involved the use of Sprague–Dawley male rats weighing 250–300 g. Animals received humane care in compliance with the European Union Directive 2010/63/EU and the Italian Legislative Decree 26/2014. Experiments were performed in compliance with the 3R principles (56, 57).

An “*a priori*” power analysis was carried out to estimate the minimum number of rats needed to reliably detect the expected effect size (55–57). Given the well-known negative effects of reperfusion on mitochondrial oxidative phosphorylation, ATP lung content was used as reference variable to explore the effects exerted by T3. Sample size calculation was based on the following assumptions: (1) number of groups: 6; (2) statistical test: one-way ANOVA; (3) expected effect size: 0.45; (4) expected standard deviation of residuals: 0.20; (5) desired power: 0.80; (6) alpha error: 0.05. The analysis provided a minimum number of 5 lungs for each experimental group. The final number of cases needed to perform the study was therefore 30 lungs.

### Ischemia model, EVLP protocol, and lung function assessment during reperfusion

Protocols for anesthesia induction and surgical procedures are provided in Supplementary Methods 1.3. To investigate the effects of T3 supplementation during EVLP, two experimental conditions were considered (Supplementary Methods 1.4): uninjured lungs (EVLP and EVLP + T3 groups), which underwent *in situ* flushing through the pulmonary artery with ice-cold Perfadex (XVIVO) and were then immediately procured; ischemic lungs (Ischemia + EVLP, and Ischemia + EVLP + T3 groups), which were subjected to 60 min-WI, in situ flushing with ice-cold Perfadex, and were then exposed to 60 min-CI before EVLP.

EVLP was performed using a custom small-animal circuit primed with Steen Solution™ (Supplementary Methods 1.5). The 180-min EVLP protocol included (1) a reperfusion phase (from 0 to 40 min), during which lungs were gradually re-warmed, ventilated and perfused, and (2) a reconditioning phase (from 40 to 180 min), during which ventilation/perfusion parameters were in a steady-state. Final tidal volume (TV) was 7 mL/kg, pulmonary artery flow (PAF) was 6 mL/min/g of predicted lung weight.

During the first 15 min, the outflow perfusate was collected from the reservoir and removed from the circuit every 5 min. At 20 min of EVLP, the reservoir was opened to allow recirculation of the perfusate.

Ventilation was initiated at 32 °C using a protective strategy with the following parameters: positive end-expiratory pressure (PEEP) 3 cmH₂O, respiratory rate 30 breaths/min, initial TV 5 mL/kg. Lungs were ventilated with FiO_2_ 40%. Recruitment maneuvers (RM) were performed before procurement and repeated at 45 and 180 min during EVLP.

Lung function was continuously assessed by monitoring airway pressures and pulmonary artery pressure. Dynamic compliance and pulmonary vascular resistance (PVR) were calculated to evaluate mechanical and vascular performance (Supplementary Methods 1.5.1).

### Sample collection

Perfusate samples were collected at regular time intervals for gas analyses and subsequent biomolecular evaluations. More specifically, during the initial 15 min of EVLP, the entire volume of Steen Solution™ passing through the pulmonary circulation was collected from the reservoir and defined as wash-out (WO). Additional 5 mL perfusate samples were collected at 60, 120, and 180 min of EVLP, processed as described in Supplementary Methods 1.6, and stored at −80 °C for subsequent biomolecular analyses.

Tissue biopsies were collected at different time points depending on the experimental group. Lungs from the Native group were immediately sectioned for biopsy collection, whereas lungs from the Ischemia group were sampled after 1 h of WI followed by 1 h of CI. Lungs from the groups subjected to EVLP (EVLP, EVLP + T3, Ischemia + EVLP, and Ischemia + EVLP + T3) were processed after 3 h of perfusion. In all experimental groups, tissue samples were collected according to a standardized sequence and each anatomical region was allocated to a predefined biomolecular assay. Tissue biopsies were snap-frozen in liquid nitrogen and stored at −80 °C for subsequent analysis.

### Outcome measures

#### Analysis of perfusate samples

Perfusate samples were used to assess the following outcomes: oxidative DNA damage, determined by quantification of 8-hydroxy-2′-deoxyguanosine (8-OHdG) concentration by immunoassay (Supplementary Methods 1.6.1); mtDNA release by droplet digital PCR (ddPCR) (Supplementary Methods 1.6.2); pulmonary cell injury (Supplementary Methods 1.6.3), by measuring adenylate kinase (AK) release using a bioluminescent assay and evaluating caspase-cleaved cytokeratin 18 (ccK18) concentration using an immunoassay.

#### Tissue analysis

Lung tissue samples were adequately processed to evaluate the following outcomes: mtDNA copy number by ddPCR (Supplementary Methods 1.7.1); tissue ATP content using a luciferase-based assay (Supplementary Methods 1.7.2); cell energetics by quantifying adenine nucleotides (ATP, ADP, AMP) using high-performance liquid chromatography (HPLC) (Supplementary Methods 1.7.3); selected gene expression analysis (88 gene of interest and 5 reference genes, Supplementary Table 1) by real-time PCR using RT^2^ Profiler PCR Arrays (Qiagen, Hilden, Germany) (Supplementary Methods 1.7.4).

#### Statistical analysis

Data are presented as mean ± standard error of the means (SEM) or median [25th-75th percentile] when appropriate. For non-normally distributed data, non-parametric tests were applied whenever appropriate. When repeated measures (RM) analyses were required, data were rank-transformed before statistical analysis.

Differences across experimental groups were investigated using one-way analysis of variance (ANOVA), two-way ANOVA, or two-way RM ANOVA, followed by Tukey’s *post-hoc* test for multi-comparison procedures. Mixed-effects models were used for longitudinal analyses when one or more repeated measurements were missing. A probability value of *p* < 0.05 was considered significant.

Associations between perfusate mtDNA copy numbers and AK, ccCK18, and 8-OHdG concentrations were assessed using Spearman’s rank correlation. The same approach was adopted to investigate potential correlations between lung mtDNA and tissue energetics.

Overall transcriptional patterns across experimental groups were examined by exploratory principal component analysis (PCA) and unsupervised hierarchical clustering, both performed using the expression data from the entire set of analyzed genes. Clustering was performed using Euclidean distance and average linkage. An additional supervised clustering analysis was conducted using only differentially-expressed genes (defined as fold change≥1.5 combined with a *p* < 0.05) to illustrate expression patterns across experimental groups.

Statistical tests were performed using the following tools: SigmaStat software 11.0 (Systat Software Inc., San Jose, CA, USA), JMP Pro 19 (JMP Statistical Discovery LLC, SAS Campus Drive, NC, USA), Prism 9.4.1 (GraphPad Software LL, La Jolla, CA, USA), and MetaboAnalyst 6.0.^1^

## Results

### Lung function during EVLP, perfusate gas partial pressures and electrolyte composition

The parameters continuously monitored during EVLP are shown in Figure 1, whereas average values measured at each time interval are reported in Supplementary Table 2. Based on our EVLP protocol, pulmonary artery flow (PAF) was gradually increased over the first 40 min of reperfusion. Therefore, the rise of mean pulmonary artery pressure (mPAP) observed in this timeframe could be explained by the progressive increase in PAF (*p*-value for time factor: *p* < 0.001 in all the experimental groups, Figure 1A). Lungs exposed to ischemia (Ischemia + EVLP and Ischemia + EVLP + T3) showed higher mPAP compared to those immediately perfused after procurement (EVLP and EVLP + T3, *p* < 0.001). T3 supplementation was associated with lower mPAP values in the EVLP + T3 group compared to the EVLP control group (*p* < 0.001), whereas no differences were found between the Ischemia + EVLP + T3 and Ischemia + EVLP groups (*p* = 0.930). This result was consistent with PVR evaluation, indicating reduced pulmonary resistance in non-ischemic lungs receiving T3 compared to the control group (*p* = 0.036, Figure 1B). Conversely, similar to mPAP, PVR values were comparable between the Ischemia + EVLP + T3 and the Ischemia + EVLP groups (*p* = 0.849).

**Figure 1:**
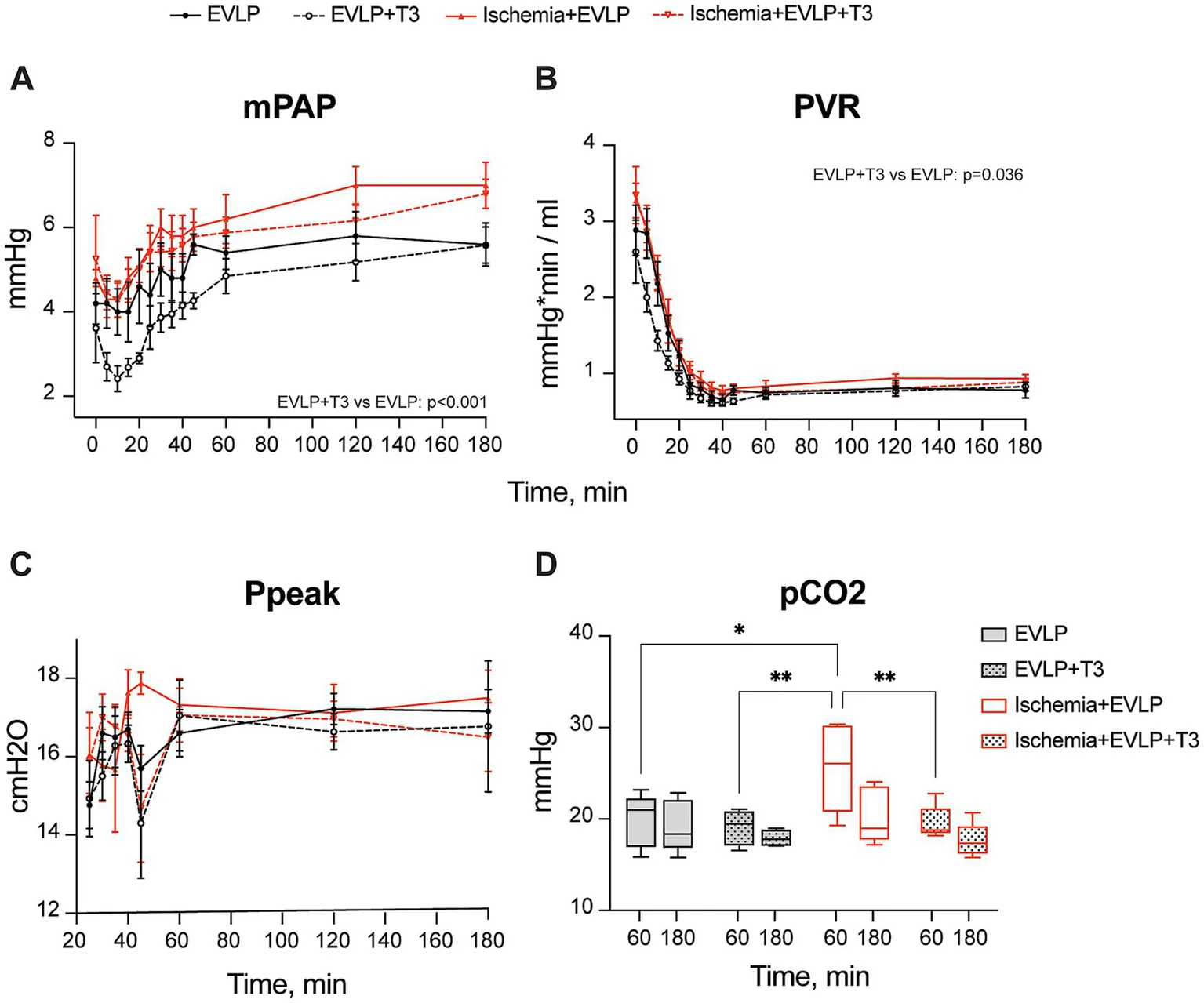
Effects exerted by T3 supplementation during EVLP on pulmonary hemodynamics, airway pressure, and gas exchange. **(A)** Mean pulmonary artery pressure (mPAP), **(B)** pulmonary vascular resistance (PVR), and **(C)** peak airway pressure (Ppeak) measured throughout EVLP. Uninjured lungs treated with T3 showed reduced mPAP (*p* < 0.001) and PVR (*p* = 0.036) compared to the EVLP group. Data are expressed as mean ± SEM, *n* = 5. Two-way RM ANOVA, Tukey’s *post-hoc* test. **(D)** Perfusate pCO at 60 and 180 min of EVLP. At 60 min, pCO2 was significantly higher in ischemic compared to uninjured lungs (*p* = 0.015). Ischemic organs receiving T3 treatment exhibited lower pCO values relative to the Ischemia + EVLP group (*p* = 0.010). Box plots indicate median (middle line), 25th, 75th percentile (box), *n* = 5. Two-way ANOVA, Tukey’s *post-hoc* test. *p < 0.05. EVLP, ex vivo lung perfusion; mPAP, mean pulmonary artery pressure; pCO_2_, partial pressure of carbon dioxide; Ppeak, peak airway pressure; PVR, pulmonary vascular resistance; T3, triiodothyronine.

With regard to airway parameters, no significant treatment-dependent differences were revealed in peak airways pressures (Ppeak) (*p* = 0.214, Figure 1C). Of note, while Ppeak did not improve in the Ischemia + EVLP group following the RM at 45 min of EVLP (*p* = 0.074), the Ischemia + EVLP + T3 group showed comparable values relative to uninjured organs (EVLP and EVLP + T3). No differences in lung compliance measured at 45 and 180 min of EVLP were detected across experimental groups (*p* = 0.763, Supplementary Figure 3).

Perfusate pO2 and pCO2 collected upstream (PRE) and downstream (POST) of the lung at 60 min and 180 min of EVLP are shown in Table 1. Overall, lungs from all experimental groups maintained physiological gas exchange capacity throughout the perfusion. Oxygen partial pressure was similar between uninjured and ischemic lungs; consistently, no effects of T3 on oxygenation capacity were observed (*p* = 0.122). Concerning CO2, an effective elimination of this gas was observed during EVLP in all the experimental groups. However, at 60 min of EVLP, pCO2, measured downstream of the lungs, was significantly higher in ischemic compared to uninjured lungs (*p* = 0.015, Figure 1D). Of note, ischemic organs receiving T3 treatment exhibited similar pCO₂ values relative to those observed in lungs immediately perfused after procurement, and significantly lower to those observed in the Ischemia + EVLP group (*p* = 0.010, Figure 1D).

**Table 1:** Perfusate gas concentrations measured upstream (PRE) and downstream (POST) of the lung at 60 min and 180 min of EVLP.

**Group**	**Time (min)**	**Sampling site**	**pO2 (mmHg)**	**pCO2 (mmHg)**
**EVLP**				
	**60**	PRE	107.9±19.3	32.1±0.9
		POST	513.4±34.6	20.7±1.2
	**180**	PRE	107.6±20.3	28.8±1.2
		POST	507.2±16	20±1.2
**EVLP+T3**				
	**60**	PRE	104.8±7.5	30.6±2.5
		POST	480.6±29.1	22.1±1.6
	**180**	PRE	97.3±9.9	26.8±1.4
		POST	461.6±38.9	20.6±1.2
**Ischemia+EVLP**				
	**60**	PRE	94.8±4.8	33.7±1.4
		POST	475.2±72.1	26.4±2.1
	**180**	PRE	93.8±3.5	32.1±1.6
		POST	494.6±47.5	24±2.5
**Ischemia+EVLP+T3**				
	**60**	PRE	106.7±7.6	26.8±0.6
		POST	464.4±45.9	20.4±0.8
	**180**	PRE	100±5.3	25.1±1
		POST	476.4±25.6	18.4±0.8

Data are expressed as mean±SEM. EVLP, ex vivo lung perfusion; PRE, perfusate collected upstream of the lung; POST, perfusate collected downstream of the lung; T3, 3, 3′, 5-triiodothyronine.

Perfusate electrolyte composition is shown in Supplementary Table 3. At wash-out and at 1 h of EVLP, untreated ischemic lungs showed higher K+ concentration compared to both the EVLP (*p* < 0.006 and *p* = 0.009, respectively) and the EVLP + T3 group (p < 0.006 and *p* < 0.001, respectively). At 1 h, lungs from the Ischemia + EVLP + T3 exhibited a modest but significant reduction in K+ release compared to the Ischemia + EVLP (*p* = 0.026, Supplementary Figure 4). The perfusate drained from the lungs exposed to ischemia showed a lower pH compared with uninjured groups (*p* = 0.015), whereas pH values in the Ischemia + EVLP + T3 were significantly higher (*p* = 0.003 vs. Ischemia + EVLP) and similar to those of uninjured lungs (*p* = 0.160 vs. EVLP and *p* = 0.901 vs. EVLP + T3). T3 supplementation was likewise associated with lower Ca++ concentration (*p* < 0.001 and *p* = 0.004 in non-ischemic and ischemic lungs, respectively) and higher base excess compared with the untreated groups at both 1 h (*p* < 0.001) and 3 h (*p* < 0.001). Finally, glucose and lactate perfusate concentrations are shown in Supplementary Table 4.

### Oxidative damage and pulmonary cell viability

Lungs exposed to ischemia showed higher 8-OHdG concentrations immediately after the onset of perfusion, indicating early oxidative DNA damage (Figure 2A). In contrast, in perfusates drained from uninjured lungs (EVLP and EVLP + T3), 8-OHdG became measurable only from 60 min of EVLP. In addition, ischemic lungs showed higher overall concentration compared to immediately perfused organs (*p* = 0.006, Figure 2A). Notably, T3 treatment was associated with a trend toward increased 8-OHdG release across both non-ischemic and ischemic conditions, although this effect did not reach statistical significance (*p* = 0.070).

**Figure 2:**
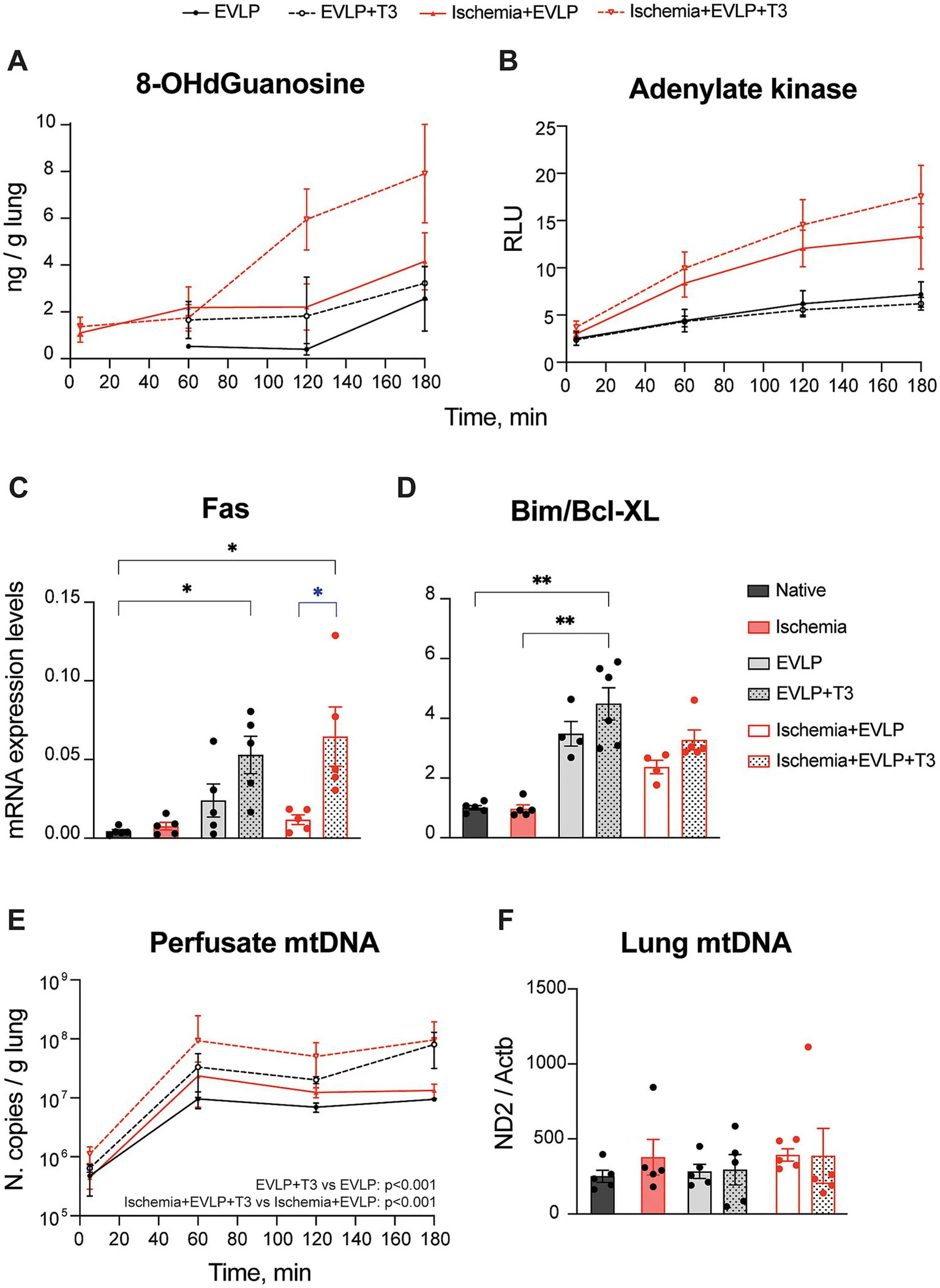
Evaluation of pulmonary cell oxidative damage and viability in lungs subjected to T3 treatment. **(A)** Perfusate concentration of the oxidative marker 8-OHdG increased over the EVLP procedure (*p* < 0.001) and was more pronounced in ischemic lungs compared to uninjured organs (*p* = 0.006). Data are expressed as mean ± SEM, *n* = 5. Two-way RM ANOVA, Tukey’s *post-hoc* test. **(B)** The release of adenylate kinase into the perfusate progressively increased over the EVLP procedure (*p* < 0.001), with higher values observed in lungs exposed to ischemia (*p* < 0.001). Data are expressed as mean ± SEM, *n* = 5. Two-way RM ANOVA, Tukey’s *post-hoc* test. **(C,D)** illustrate selected gene expression analysis of lung homogenates. Compared to native lungs, in T3-treated lungs, there was induction of Fas death receptor, irrespective of ischemic challenge (EVLP + T3 vs. Native, *p* = 0.026; Ischemia + EVLP + T3 vs. Native, *p* = 0.014). Moreover, the EVLP + T3 group was associated with an increase in the Bim/Bcl-XL ratio(*p* = 0.005 vs. Native). Bars denote mean± SEM, *n* = 5. One-way ANOVA, Tukey’s *post-hoc* test. *p < 0.05, **p < 0.01. Exploratory two-way ANOVA, followed by Tukey’s *post-hoc* test, was subsequently performed considering only perfused lungs to evaluate the effects of ischemia and treatment: blue asterisk denotes *p*-value for T3-dependent effect, *p < 0.05. **(E)** T3 treatment was associated with higher release of mtDNA in both uninjured and ischemic lungs compared with control groups (*p* < 0.001 in both conditions). Data are expressed as mean ± SEM, *n* = 5. Two-way RM ANOVA, Tukey’s *post-hoc* test. **(F)** Lung tissue mtDNA copy number was similar across experimental groups. Bars denote mean ± SEM, *n* = 5. One-way ANOVA, *p* = 0.467. 8-OHdG, 8-hydroxy-2′-deoxyguanosine; EVLP, ex vivo lung perfusion; mtDNA, mitochondrial DN A; T3, triiodothyronine.

We investigated pulmonary cell viability by assessing the release of cell death biomarkers in the perfusate throughout the EVLP procedure. Adenylate kinase (AK) concentration increased over the EVLP procedure in all the experimental groups (*p*-value for time factor: *p* < 0.001, Figure 2B). Compared to uninjured lungs, organs exposed to ischemia exhibited a higher release of this factor (*p* < 0.001). No differences were found between the T3-treated and the control groups (*p* = 0.698 in uninjured lungs and *p* = 0.261 in ischemic lungs). The epithelial cell death biomarker ccCK18 showed a similar trend, with increasing concentration over time (*p*-value for time factor: *p* < 0.001), and comparable release in organs treated with T3 relative to untreated lungs (*p* = 0.377 in uninjured organs and *p* = 0.051 in ischemic lungs, Supplementary Figure 5).

Based on these findings, we evaluated the expression of a panel of genes involved in cell death (Supplementary Table 1). This analysis revealed a significant upregulation of *Fas* transcript compared to baseline in lungs subjected to T3 treatment, both in the absence of ischemic challenge and following exposure to ischemia (One-way ANOVA: EVLP + T3 vs. Native, *p* = 0.026; Ischemia + EVLP + T3 vs. Native, *p* = 0.014; Two-way ANOVA: Ischemia + EVLP + T3 vs. Ischemia + EVLP, *p* = 0.037; Figure 2C). Moreover, a higher *Bim/Bcl-XL* ratio was observed in the EVLP + T3 group compared to native lungs (*p* = 0.005, Figure 2D).

To investigate oxidative stress-related mitochondrial damage, the release of mtDNA was assessed in the perfusates. T3 supplementation was associated with higher copy number of mtDNA in both EVLP + T3 and Ischemia + EVLP + T3 groups, compared with the corresponding control groups (*p* < 0.001 in both conditions, Figure 2E). Conversely, mtDNA assessment in lung homogenates revealed similar tissue abundance across experimental groups (*p* = 0.467, Figure 2F).

Potential associations between mtDNA and the release of 8-OHdG, AK, and ccCK18 were likewise explored. Perfusate mtDNA levels positively correlated with both AK (*r* = 0.538, 95% CI 0.349–0.686; *p* < 0.0001; Supplementary Figure 6) and ccCK18 (*r* = 0.565, 95% CI 0.380–0.706, p < 0.0001; Supplementary Figure 7). Conversely, no significant association was found between mtDNA and 8-OHdG perfusate concentrations (*r* = 0.039, 95% CI -0.259–0.331, *p* = 0.793; Supplementary Figure 8).

### Lung cell energetic status

Pulmonary cell energetics was evaluated in lung homogenates using a bioluminescence-based method, as previously reported (58). Exposure to ischemia was associated with lower tissue ATP content compared with the EVLP group (Ischemia vs. EVLP: *p* = 0.047; Ischemia + EVLP vs. EVLP: *p* = 0.009, Figure 3A). T3 treatment did not restore ATP content in ischemic lungs (Ischemia + EVLP + T3 vs. Ischemia + EVLP: *p* = 0.999). Exploratory two-way ANOVA restricted to the perfused lungs confirmed that ischemic exposure was associated with ATP depletion (Ischemia + EVLP vs. EVLP: *p* = 0.002). This analysis likewise indicated that T3 supplementation in non-ischemic lungs significantly limited the increase in ATP associated with the EVLP procedure (EVLP + T3 vs. EVLP: *p* = 0.029).

**Figure 3:**
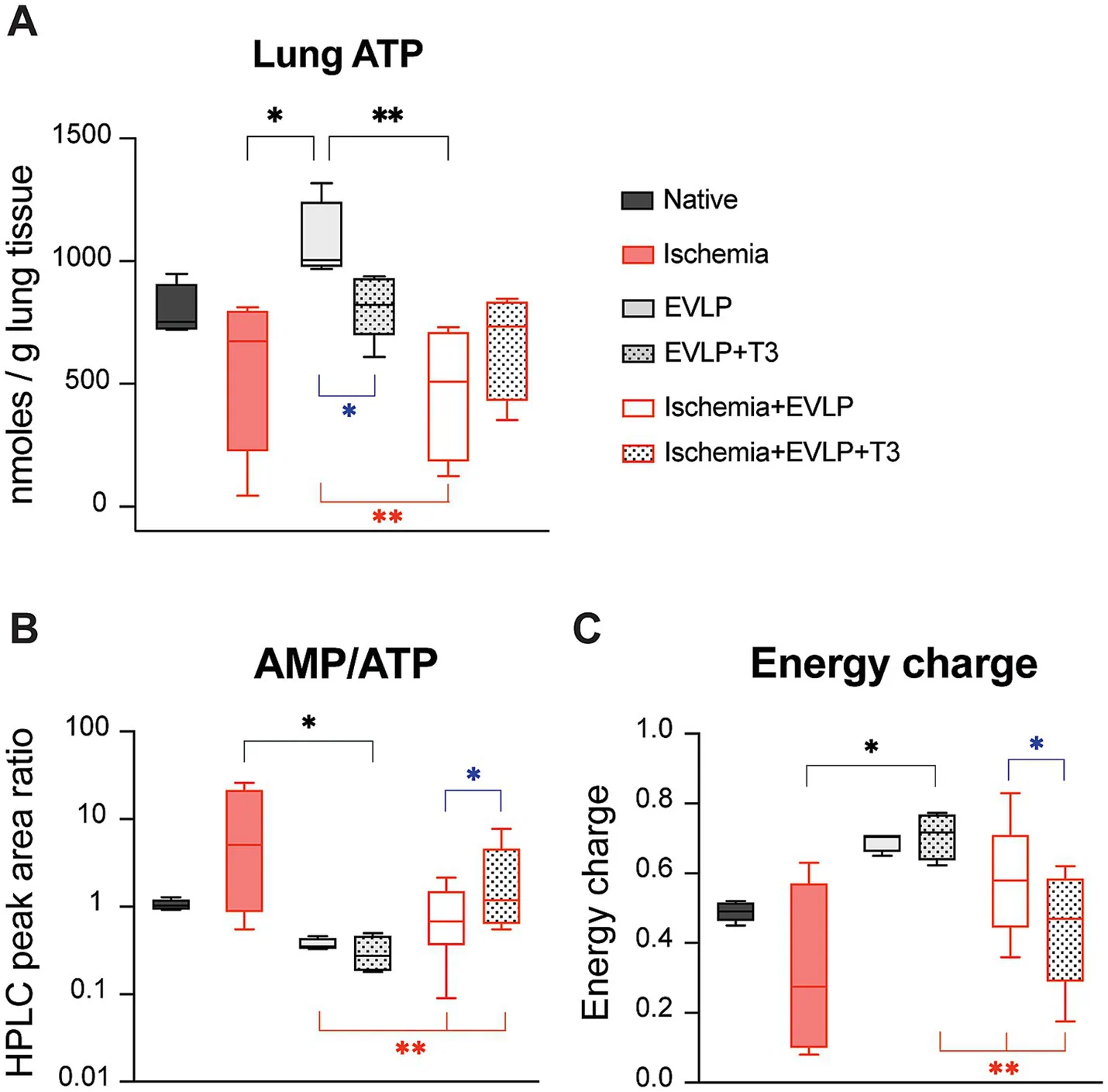
Effects exerted by T3 supplementation during EVLP on pulmonary energetic status. **(A)** Tissue ATP content measured by luciferase-based bioluminescence in lung homogenates. Ischemic exposure was associated with lower ATP content in both non-perfused (*p* = 0.047) and perfused lungs (*p* = 0.009). T3 supplementation during EVLP did not restore ATP content in ischemic lungs (*p* = 0.999). Bars denote mean ± SEM, *n* = 5. One-way ANOVA, Tukey’s *post-hoc* test. *p < 0.05, **p < 0.01. Exploratory two-way ANOVA, followed by Tukey’s *post-hoc* test, was subsequently performed considering only perfused lungs to evaluate the effects of ischemia and treatment: red asterisk denotes *p*-value for ischemia-dependent effect, **p < 0.01; blue asterisk denotes *p*-value for T3-dependent effect, *p < 0.05. **(B)** AMP/ATP ratio determined in lung homogenates by HPLC. Ischemic lungs showed a marked increase in AMP/ATP ratio, indicating energetic stress. T3 treatment during EVLP of lungs exposed to ischemia was associated with a higher AMP/ATP ratio than matched untreated controls. Box plots illustrate median (middle line), 25th and 75th percentile (box), *n* = 5. One-way ANOVA, Tukey’s *post-hoc* test: black asterisk denotes *p*-value for overall group differences, *p < 0.05. Exploratory two-way ANOVA, followed by Tukey’s *post-hoc* test, was subsequently performed considering only perfused lungs to evaluate the effects of ischemia and treatment: ed asterisk denotes *p*-value for ischemia-dependent effect, **p < 0.01; blue asterisk denotes *p*-value for T3-dependent effect, *p < 0.05. **(C)** Cellular energy charge calculated based on adenine nucleotide levels determined in lung homogenates by HPLC. T3 supplementation during EVLP did not improve the energetic state in ischemic lungs and was associated with lower energy charge values compared with the Ischemia + EVLP. Box plots illustrate median (middle line), 25th and 75th percentile (box), *n* = 5. One-way ANOVA, Tukey’s *post-hoc* test: lack asterisk denotes *p*-value for overall group differences, *p < 0.05. Exploratory two-way ANOVA, Tukey’s *post-hoc* test was subsequently performed considering only perfused lungs to evaluate factor effects: red asterisk denotes *p*-value for ischemia-dependent effect, **p < 0.01; blue asterisk denotes *p*-value for T3-dependent effect, *p < 0.05 (AMP, adenosine monophosphate; ATP, adenosine triphosphate; EVLP, ex vivo lung perfusion; T3, triiodothyronine).

To further characterize these observations, HPLC analysis was performed on the same samples to assess ATP derivative concentrations. These data were then used to calculate AMP/ATP ratios as well as pulmonary cell energy charge using a validated formula (59). Consistent with the results provided by pulmonary ATP determination, lungs exposed to ischemia alone showed a higher AMP/ATP ratio compared with uninjured lungs subjected to EVLP (Ischemia vs. EVLP: *p* = 0.045, Figure 3B). To better explore the effect of T3 treatment, a two-way ANOVA analysis was performed considering only the perfused groups. While confirming that ischemia challenge was associated with a deteriorated energy status (Ischemia + EVLP vs. EVLP: *p* = 0.005; Ischemia + EVLP + T3 vs. EVLP: *p* = 0.005), the analysis disclosed a significant further increase in AMP/ATP ratio in ischemic lungs receiving T3 supplementation (Ischemia + EVLP + T3 vs. Ischemia + EVLP: *p* = 0.034, Figure 3B). Evaluation of pulmonary energy charge confirmed these results, a further reduction of cell energetic pool in ischemic lungs treated with T3 (Ischemia + EVLP + T3 vs. Ischemia + EVLP: *p* = 0.028, Figure 3C).

### Pulmonary expression of selected genes involved in inflammation, response to stress, and survival

We investigated the expression of 88 genes involved in the following functional categories (Supplementary Table 1): (1) inflammation and innate immunity, (2) mitochondrial energy metabolism, (3) oxidative stress and antioxidant response, (4) stress response and survival, (5) cell death, (6) cell death—mitochondrial pathway, (7) growth and differentiation.

Global differences across experimental groups were explored by PCA. As shown in Figure 4A, a main distinction was observed along the PC1 axis between the non-perfused groups, including Native and ischemic lungs, and the groups subjected to the EVLP procedure. Among the perfused lungs, the analysis discriminated between uninjured (EVLP and EVLP + T3) and ischemic lungs (Ischemia + EVLP and Ischemia + EVLP + T3), with a partial overlap between T3-treated and untreated groups. Notably, along the PC2 axis, the EVLP + T3 group displayed an overall transcriptional profile resembling that observed in the perfused ischemic groups.

**Figure 4:**
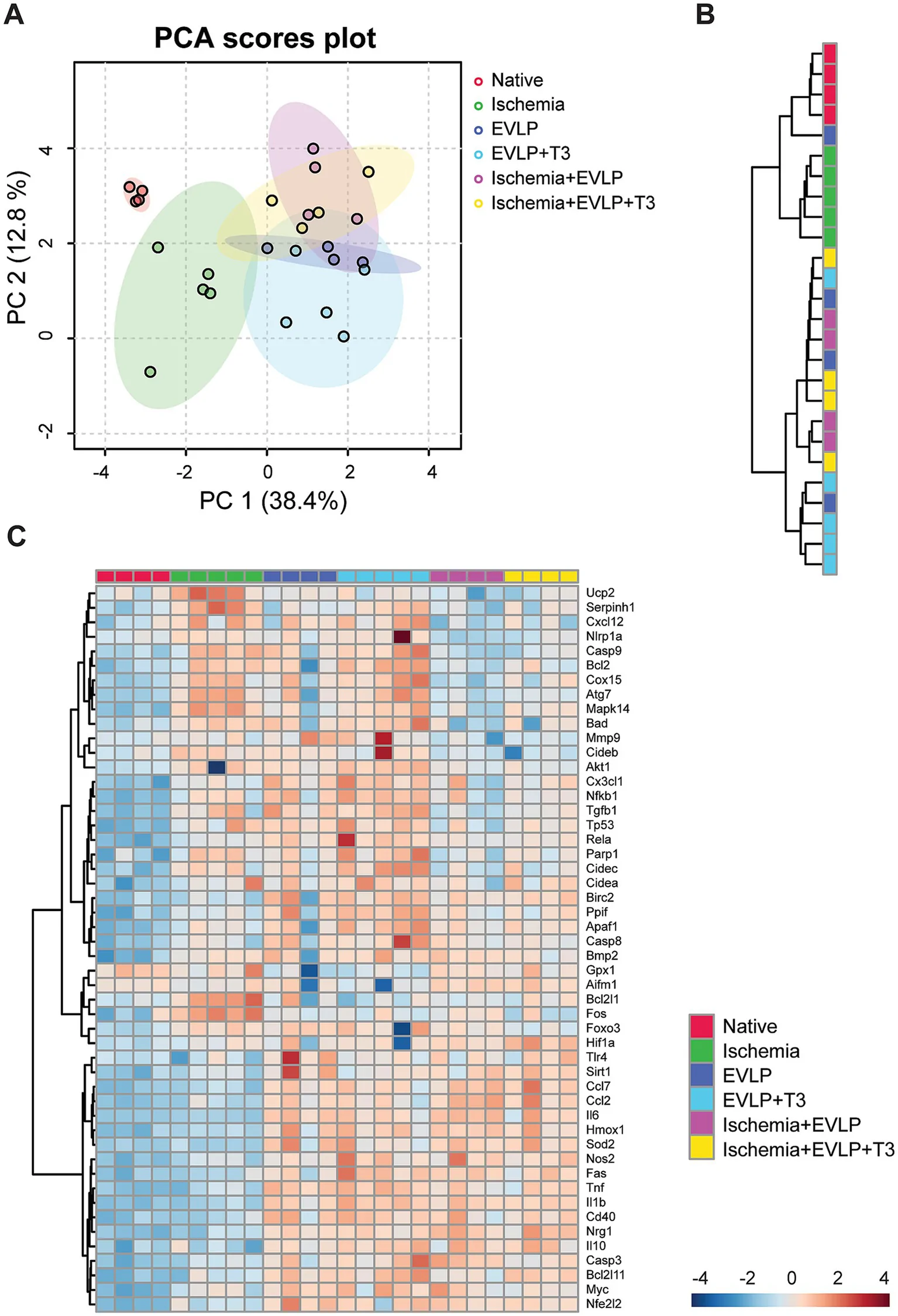
Multivariate analysis of selected gene expression in the pulmonary tissue. Total RNA was extracted from lung homogenates and the expression of a selected gene panel was analyzed using a real-time PCR-based array. **(A)** Principal component analysis (PCA) score plot of gene expression profiles obtained from lung tissue samples. Each point represents an individual sample, colored according to the experimental group. Ellipses represent group clustering. *PC1* and *PC2* explain 38.4 and 12.8% of the total variance, respectively. **(B)** Dendrogram illustrating hierarchical clustering of samples based on gene expression patterns. Colored bars denote the corresponding experimental groups. **(C)** Heatmap showing relative expression levels of significantly differentially expressed genes involved in cell death, inflammation, mitochondrial function, and stress responses. Rows represent genes and columns represent individual samples. Gene expression values are displayed as log10-transformed normalized expression values, with red indicating higher expression and blue indicating lower expression relative to the mean. Colored bars indicate the corresponding experimental groups. EVLP, ex vivo lung perfusion; PCA, principal component analysis; T3, triiodothyronine.

Unsupervised hierarchical clustering was consistent with PCA findings, revealing a distinctive clustering between non-perfused lungs and organs subjected to EVLP (Figure 4B). Of note, T3-treated and untreated lungs did not segregate into separate clusters.

The heatmap reported in Figure 4C illustrates the log10-transformed normalized expression values of differentially expressed genes across experimental groups. A distinctive EVLP-dependent inflammatory profile was observed in both uninjured and ischemic lungs. Compared to the corresponding control groups, T3 supplementation was associated with significant changes in gene expression. The functional categories enriched among the T3-modulated genes (upregulated by T3 vs. untreated controls irrespective of ischemia) were “cell death—mitochondrial pathway” (6/11 genes), “stress response and survival” (7/12 genes), and “inflammation and innate immunity” (7/22 genes). Average gene expression values of each experimental group, together with *p*-values provided by both one-way and two-way ANOVA analysis, are reported in Supplementary Table 5.

## Discussion

The present study investigated whether T3 supplementation during EVLP could mitigate IR-induced molecular injury while improving lung function and cell energetics. Although T3-treated lungs showed subtle improvements in selected functional parameters, T3 administration did not counterbalance the ischemia-induced molecular damage in pulmonary cells. Conversely, treatment was associated with increased mtDNA release into the perfusate and higher AMP/ATP ratios, without reducing biomarkers of cell injury or oxidative DNA damage. These findings suggest that, T3-mediated metabolic stimulation during EVLP does not promote energetic recovery and could instead exacerbate mitochondrial stress.

The repair of marginal organs represents a major goal in transplantation research, since a substantial proportion of lungs is still declined due to poor quality (60). IR injury has a central role in determining organ quality, since it triggers a complex cascade of deleterious biological events, leading to increased vascular permeability, alveolar damage, and pulmonary edema (38, 61). These processes significantly contribute to the development of PGD (1), which could be associated with CLAD (2, 3), and were also reported to promote the induction of donor-specific alloimmunity (62). In this context, the effectiveness of EVLP to repair pre-existing damage appears limited (4). Moreover, increasing evidence has indicated that a certain degree of injury could occur during the perfusion procedure itself (21, 22). Therefore, coupling EVLP with therapeutic strategies able to reduce the detrimental consequences of IR could significantly enhance its reconditioning potential.

The present research investigated the effects of T3 supplementation in Steen Solution™ during experimental EVLP. The rationale was based on the multiple positive effects exerted by THs in lung injury models (41, 46–51). In particular, the ability of T3 to support mitochondrial function and mitigate cell energy failure (41, 46, 52, 63–67) are especially promising in the context of IR (33, 34). However, our findings indicate that these beneficial properties do not necessarily translate to the isolated ischemic lung.

From a functional perspective, T3 supplementation reduced pulmonary vascular resistance, consistent with its known vasodilatory and hemodynamic effects (68). In addition, an improved carbon dioxide clearance was observed in ischemic lungs subjected to T3 treatment. However, these modest functional changes were not coupled with molecular evidence of protection, indicating a possible dissociation between macroscopic parameters and subcellular injury, a concept that has been already discussed in previous studies involving EVLP (7, 10, 69–72). In the present research, T3 supplementation was associated with a significant increase in mtDNA release in both uninjured and ischemic lungs, together with a trend toward higher 8-OHdG perfusate concentrations. These observations are consistent with a study by Cioffi and coworkers, who reported increased mtDNA copy number and higher plasma 8-OHdG after T3 administration in resting rats (63). Since ROS-induced mitochondrial damage and subsequent mtDNA release are key events underlying pulmonary cell injury in the setting of lung transplantation (73, 74), these results suggest that T3 increased mitochondrial instability, rather than mitigating ischemia-induced dysfunction.

The assessment of pulmonary cell viability and energetics further supported this hypothesis. Indeed, T3 supplementation was associated with increased *Fas* expression in ischemic lungs, likely reflecting apoptotic priming within the mitochondrial pathway (75). Additionally, ischemic lungs exposed to T3 showed higher AMP/ATP ratios and a lower cellular energy charge compared to the untreated group. These results suggest an enhanced mitochondrial stress in T3-treated lungs, potentially associated with impaired ATP production (76). Finally, although gene expression analysis revealed only a modest transcriptional modulation associated with T3 treatment, the observed changes support a shift toward pro-death signaling rather than a survival-promoting effect.

These findings should be interpreted considering the broad and potent effects exerted by THs on mitochondrial function. Under physiological conditions, these hormones promote cellular oxygen consumption by stimulating mitochondrial respiration (40). Although these actions are vital in healthy cells, increasing metabolic flux in cells challenged by ischemia and nutrient depletion can paradoxically exacerbate oxidative stress and mitochondrial dysfunction (76). In line with this concept, seminal studies in hyperthyroid animal models demonstrated that excessive TH signaling can enhance mitochondrial ROS production and increase susceptibility to IR injury (77, 78). On the other hand, more recent research showed that restoration of physiologic TH signaling after ischemic injury could support mitochondrial function and tissue recovery (65, 66, 79, 80), even in the context of ex situ perfusion (53). Taken together, these apparently contradictory observations indicate that the effects of T3 are highly context-dependent and likely influenced by hormone dose, timing of administration, and by the metabolic state of the target tissue (81).

Of interest, another important factor to consider is the hormonal background of albumin-based perfusion solutions, such as Steen Solution™ (82–84). In fact, these pharmaceutical preparations can carry co-purified proteins and small molecules from donor plasma (85), including THs that bind albumin with high affinity (86). In the present study, both lots of Steen Solution™ used for EVLP contained measurable amounts of T3 and thyroxine (T4) (Supplementary Table 6). These observations collectively highlight the need for careful consideration when evaluating THs-based interventions in EVLP.

Our study shows some limitations. First, only one T3 dose and one timing of administration were tested; therefore, we cannot exclude that different exposure regimens could have induced different biological effects. In particular, T3 supplementation may have different effects throughout the ischemic time before the reperfusion as a preconditioning strategy. Second, as indicated by gene expression analyses, although the combined warm and cold ischemia model reproduces clinically relevant aspects of DCD lung injury, it also introduced biological heterogeneity that may have contributed to data variability. Thus, the possibility that subtle effects of T3 could have remained undetected cannot be entirely excluded. Third, only male rats were used, since at the time the experiments were performed, no sex-based differences were reported in the context of short-term organ ex situ perfusion. Finally, since a rat EVLP model was used, caution is required when extrapolating the results to clinical lung perfusion. Nevertheless, the consistency across mtDNA release, oxidative damage, apoptotic signaling, and energetic impairment supports the biological robustness of our observations.

In conclusion, under the specific experimental conditions adopted in the present study, T3 supplementation during EVLP did not support molecular recovery in ischemic lungs, despite modest improvements in selected pulmonary function parameters. On the contrary, T3 treatment appeared to be associated with amplified mitochondrial stress signals and with impaired cell energetics. These observations suggest that this approach could lead to additional metabolic demand that becomes maladaptive when administered under conditions of oxygen and nutrient deprivation. Therefore, therapeutic strategies aimed at enhancing cell metabolism during EVLP should be carefully balanced against the bioenergetic state of the organ. These conclusions are limited to the specific T3 exposure and experimental conditions evaluated in the present study; the effects of alternative dosing and administration regimens remain to be established.

## Data Availability

The data supporting the findings of this study are presented in the article and its Supplementary material. Further inquiries can be directed to the corresponding author/s.

## Footnotes

1
https://www.metaboanalyst.ca/home.xhtml

## Glossary

8-OHdG: 8-hydroxy-2′-deoxyguanosine
AK: adenylate kinase
AMP: adenosine monophosphate
ANOVA: analysis of variance
ATP: adenosine triphosphate
ccK18: caspase-cleaved cytokeratin 18
CI: cold ischemia
CLAD: chronic lung allograft dysfunction
ddPCR: droplet digital PCR
EVLP: ex vivo lung perfusion
FiO₂: fraction of inspired oxygen
HPLC: high-performance liquid chromatography
IRI: ischemia/reperfusion injury
mPAP: mean pulmonary artery pressure
mtDNA: mitochondrial DNA
PAF: pulmonary artery flow
PCA: principal component analysis
pCO₂: partial pressure of carbon dioxide
PEEP: positive end-expiratory pressure
PGD: primary graft dysfunction
Ppeak: peak airway pressure
PVR: pulmonary vascular resistance
RM: recruitment maneuver
ROS: reactive oxygen species
T3: triiodothyronine
TH: thyroid hormone
TV: tidal volume
WI: warm ischemia
