# Supplementary material for: Effects of 3,3′,5-triiodothyronine supplementation during ex vivo lung perfusion on ischemia/reperfusion injury in rat lungs
Source: Front Med (Lausanne). 2026 Sep 10;13:1924438. doi: 10.3389/fmed.2026.1924438 (PMC13625183; doi:10.3389/fmed.2026.1924438)
Supplement: Supplementary file 1 [file Supplementary_file_1.docx]

Supplementary material

TITLE: ARTICLE Effects of 3,3’,5-triiodothyronine (T3) supplementation during ex vivo lung perfusion on ischemia/reperfusion–associated cell injury in rat lungs

Table of Contents

[1. Supplementary methods	3](#_Toc236395968)

[1.1. Experimental design and treatment randomization scheme	3](#_Toc236395969)

[1.2. T3 treatment preparation	3](#_Toc236395970)

[1.3. Surgical procedures	3](#_Toc236395971)

[1.4. Lung procurement, ischemia induction, and in situ flushing	4](#_Toc236395972)

[1.4.1. Uninjured lungs: EVLP and EVLP+T3 groups	4](#_Toc236395973)

[1.4.2. Ischemic lungs: Ischemia, Ischemia+EVLP, and Ischemia+EVLP+T3 groups	4](#_Toc236395974)

[1.5. Ex vivo lung perfusion	5](#_Toc236395975)

[1.5.1. Lung function monitoring during EVLP	5](#_Toc236395976)

[1.6. Analysis of perfusate samples	6](#_Toc236395977)

[1.6.1. Evaluation of 8-hydroxy-2'-deoxyguanosine (8-OHdG) concentration	6](#_Toc236395978)

[1.6.2. Quantification of mitochondrial DNA (mtDNA) copy numbers in perfusate samples	6](#_Toc236395979)

[1.6.3. Cell damage markers: Caspase-cleaved keratin 18 (CK18) and Adenylate kinase (AK)	7](#_Toc236395980)

[1.7. Evaluations performed on rat lung biopsies	7](#_Toc236395981)

[1.7.1. Quantification of mtDNA copy numbers in lung tissue	7](#_Toc236395982)

[1.7.2. ATP content assessment	7](#_Toc236395983)

[1.7.3. ATP derivatives by High-performance liquid chromatography (HPLC)	8](#_Toc236395984)

[1.7.4. RNA purification and analysis of the expression of selected genes	8](#_Toc236395985)

[2. Supplementary tables	9](#_Toc236395986)

[Supplementary Table 1. List of genes assessed by real-time PCR	9](#_Toc236395987)

[Supplementary Table 2. Hemodynamics and airway parameter monitoring during EVLP	10](#_Toc236395988)

[Supplementary Table 3. Perfusate gas-analysis throughout the EVLP procedure	12](#_Toc236395989)

[Supplementary Table 4. Perfusate glucose and lactate concentration over EVLP	13](#_Toc236395990)

[Supplementary Table 5. Expression values of selected genes in lung homogenates	14](#_Toc236395991)

[Supplementary Table 6. Evaluation of Steen Solution^TM^ hormonal content	18](#_Toc236395992)

[Supplementary Table 7. Effect estimates and 95% CI for selected energetic, apoptotic, and functional outcomes.	19](#_Toc236395993)

[3. Supplementary figures	21](#_Toc236395994)

[Supplementary Figure 1. Experimental design and treatment randomization scheme.	21](#_Toc236395995)

[Supplementary Figure 2. Induction of Thyroid hormone responsive (Thrsp1) gene in lungs treated with T3	22](#_Toc236395996)

[Supplementary Figure 3. Lung compliance over the EVLP procedure	23](#_Toc236395997)

[Supplementary Figure 4. K+ release into the perfusate during EVLP	24](#_Toc236395998)

[Supplementary Figure 5. ccCK18 release into the perfusate during EVLP	25](#_Toc236395999)

[Supplementary Figure 6. Association between mtDNA and AK release in the perfusate.	26](#_Toc236396000)

[Supplementary Figure 7. Association between mtDNA and ccCK18 release in the perfusate.	27](#_Toc236396001)

[Supplementary Figure 8. Association between mtDNA and 8OHdG in the perfusate.	28](#_Toc236396002)

[Supplementary Figure 9. Association between tissue mtDNA and tissue ATP content.	29](#_Toc236396003)

[Supplementary Figure 10. Association between tissue mtDNA and tissue AMP/ATP ratio.	30](#_Toc236396004)

[4. References	31](#_Toc236396005)

# Supplementary methods

## Experimental design and treatment randomization scheme

The study used animals within predefined weight ranges to minimize variability among the experimental groups attributable to these parameters.

The randomization scheme adopted in the present study is illustrated in the Supplementary figure 1. The allocation of lungs to the EVLP or non-EVLP branches was established a priori for logistical reasons. In fact, EVLP procedure required freshly prepared reagents and sterilized consumables/ glassware, with the sterilization procedure involving incubation with specific reagents for several hours.

Within the non-EVLP branch, lungs were allocated to the Native (n=5) or Ischemia groups (n=5).

Within the EVLP branch, a first round of simple randomization was performed to assign lungs to warm ischemia (WI) followed by in situ perfusion (ISP), lung procurement, and cold ischemia (CI) or ISP alone (n=10 for each experimental condition). A second round of simple randomization was performed to allocate lungs to T3 (EVLP+T3, n=5 and Ischemia+EVLP+T3, n=5) or saline treatment (EVLP, n=5 and Ischemia+EVLP, n=5).

Treatment allocation was blinded: the allocation code was known only to the researcher responsible for preparing the treatment and was not disclosed to the researchers performing the procedures on the lungs.

## T3 treatment preparation

Treatment was performed using a commercially available formulation of synthetic T3 (Sigma-Aldrich, Merck KGaA, Darmstadt, Germany). One mg of T3 was dissolved in 1 ml NaOH 1 N and subsequently diluted with 19 ml of Steen Solution™ (XVIVO) to obtain a final concentration of 50 µg/mL. This solution was directly added to the perfusion fluid after 20 min of reperfusion. After wash-out collection, 1.36 mL of this solution, corresponding to 68 µg T3, was added to the perfusion fluid, resulting in a final concentration of 648 µg/L, equivalent to 0.99 µM.

T3 treatment was associated with induction of Thyroid hormone responsive (Thrsp1) gene (Supplementary Figure 1), confirming biological target engagement.

## Surgical procedures

All surgical procedures were performed under sterile conditions and general anaesthesia. Anaesthesia was induced with isoflurane vapor (4–5% in 1 L O₂) delivered in an induction chamber. Anaesthesia was maintained with isoflurane in combination with a single intraperitoneal injection of sodium thiopental (80 mg/kg), followed by an intravenous injection of heparin (2 IU/g).

The trachea was exposed through a cervical incision and cannulated with a 14-G tube. Mechanical ventilation with continuous positive airway pressure (CPAP) was initiated, with 100% O₂, 0.3 L/min, positive End-Expiratory Pressure (PEEP) 3 cmH₂O, spontaneous breathing. Continuous monitoring of airway pressures and volumes was likewise started.

The abdominal cavity was accessed by laparotomy (xipho-pubic incision in supine position), followed by sectioning of the inferior vena cava which caused rat euthanasia by exsanguination. This procedure marked the beginning of the WI phase for the following experimental groups: Ischemia, Ischemia+EVLP, and Ischemia+EVLP+T3.

The thoracic cavity was then accessed via a median incision and sternotomy. The thymus was removed, and the lungs were exposed after sectioning the hemidiaphragms, pulmonary ligaments, and pericardium.

A recruitment manoeuvre (RM) was performed by manually insufflating the lungs with a syringe in sequential 1 mL increments until a maximal volume of 25 mL/kg or a maximal peak airway pressure (Ppeak) of 20 cmH₂O was reached.

A cannula was inserted into the pulmonary artery through an incision in the right ventricle, carefully avoiding air embolism. The auricles and the apex of the heart were then sectioned to open the pulmonary circulation to atmospheric pressure.

Pressure-controlled ventilation was then initiated with ambient air with the following setup: PEEP 3 cmH₂O, inspiratory pressure 10 cmH₂O, respiratory rate 10 breaths/min, inspiratory/expiratory ratio 1:1.

## Lung procurement, ischemia induction, and in situ flushing

### Uninjured lungs: EVLP and EVLP+T3 groups

In these experimental groups, the pulmonary circulation was flushed through the pulmonary artery cannula with 25 mL of ice-cold Perfadex (XVIVO) solution at a pressure of 30 cmH₂O. Next, heart–lung block was immediately procured for subsequent connection to the EVLP circuit.

### Ischemic lungs: Ischemia, Ischemia+EVLP, and Ischemia+EVLP+T3 groups

In these experimental groups, lungs were exposed to 60 min of WI before pulmonary flushing. Intrathoracic temperature was continuously monitored with a probe and maintained between 29-35.5°C using a heat lamp. During WI, the lungs were kept deflated. Total WI time was calculated from the time of euthanasia to the start of flushing with Perfadex, which was performed following the same protocol used for the uninjured groups. Next, lungs were procured and placed in ice-cold Perfadex to induce 60 min- CI. During CI time, temperature was maintained 4-11°C and lungs were ventilated under CPAP, with PEEP 3 cmH₂O and FiO_2_ 40%.

## Ex vivo lung perfusion

The EVLP circuit consisted of pvc tubing, an air bubble trap, a glass chamber, a peristaltic pump, a reservoir, a membrane lung (Micro-1, Kewei Rising Medical, Shenzhen, China), a heat exchanger, and a small animal ventilator (SAR-830, CWE Incorporated, Pennsylvania, USA).

The circuit was primed with 125 mL of Steen Solution™ (XVIVO) supplemented with 800 IU heparin (Epsoclar, Pfizer Italia S.r.l., Latina, Italy) and Antibiotic-Antimycotic (Gibco, Thermo Fisher Scientific, Waltham, MA, USA) under sterile conditions.

The heart–lung block was placed on the reservoir within the glass chamber. The perfusion circuit was connected to the pulmonary artery cannula, while the tracheal cannula remained connected to the ventilation circuit. Graft temperature was continuously monitored throughout the procedure using a probe positioned between the lungs and the reservoir.

EVLP began with a protective reperfusion phase, during which the heat exchanger temperature, pulmonary artery flow (PAF), and tidal volume (TV) were gradually increased over 45 min. Target values were: temperature: 37.5±0.5 °C; TV: 7 mL/kg; PAF, 6 mL/min/g of predicted lung weight (PLW = 0.0053 × rat weight [g] − 0.48 g). Protective ventilation was initiated once graft temperature reached 32 °C, approximately 25 min after the start of reperfusion. Ventilation parameters were set at a respiratory rate (RR) of 30 breaths/min, an initial TV of 5 mL/kg, and a PEEP of 3 cmH₂O, with FiO_2_ 40%.

Achievement of these set points marked the beginning of the steady-state (reconditioning) phase, which lasted from 45 to 180 min of EVLP. During this phase, the lungs were ventilated with FiO₂ 40%, while the membrane lung was ventilated with a gas mixture containing 5% CO₂, 21% O₂, and 74% N₂. Two RM were performed at 45 and 180 min, during which PAF was transiently reduced by 50%.

### Lung function monitoring during EVLP

Airway pressure, mean pulmonary artery pressure (mPAP), Ppeak, and PEEP were continuously monitored and recorded using a pressure transduction system connected to LabChart software (Powerlab, ADInstruments, Dunedin, New Zeland). Values were recorded every 5 min until 45 min of EVLP and subsequently at 60, 120, and 180 min. Temperature, PAF, and TV were likewise recorded at these timepoints.

Lung compliance was measured after each RM. Four time points were selected: baseline (after opening of the thoracic cavity), post–WI (between warm and cold ischemia), and at 45 and 180 min of EVLP. Compliance was calculated from the plateau pressure, measured after inflating the lungs with a syringe delivering a volume equivalent to one tidal volume over approximately 1 s.

Gas exchange was evaluated at 60 and 180 min of EVLP by simultaneously sampling perfusate upstream (PRE) and downstream (POST) of the pulmonary circulation, after passage through the membrane lung. During these assessments, the lungs were ventilated with 100% O₂.

Total pulmonary vascular resistance (PVR) was calculated according to the following equation: PVR = (PAP − WP) / PAF, where wedge pressure (WP) was assumed to be equal to atmospheric pressure (0 mmHg), since the left atrium was open to the atmosphere and the zero reference for all pressure measurements was set at atmospheric pressure.

## Analysis of perfusate samples

Samples were centrifugated at 2500 rpm for 15 min at 4°C (Heraeus Multifuge X3R, Thermo Fisher Scientific Inc., Waltham, MA, USA). Supernatants were collected and stored at - 20 ◦C for subsequent evaluation of mediator concentration.

Perfusate samples were used to assess the following parameters:

### Evaluation of 8-hydroxy-2'-deoxyguanosine (8-OHdG) concentration

The release of oxidized form of 2’-deoxyguanosine was assessed as a biomarker of oxidative damage to DNA (DNA damage competitive ELISA, Thermo Fisher Scientific). Absorbance reading was performed at 450 nm using a multi-mode microplate reader (Synergy HTX).

### Quantification of mitochondrial DNA (mtDNA) copy numbers in perfusate samples

Total DNA was extracted from 200 μL of perfusate using the QIAamp DNA Mini Kit (QIAGEN, Hilden, Germany), according to the manufacturer’s instructions. DNA concentration was measured using a NanoDrop ND-1000 spectrophotometer (Thermo Fisher Scientific, Waltham, MA, USA)..

Mitochondrial DNA was quantified by droplet digital PCR (ddPCR). Each reaction had a final volume of 20 μL and contained 20 ng of total DNA, 11 μL of 2× ddPCR Supermix for Probes (Bio-Rad Laboratories, Hercules, CA, USA), 1.1 μL of PrimePCR™ ddPCR™ Expression Probe Assay targeting the mitochondrial NADH dehydrogenase subunit 2 (ND2) gene (FAM), and 1.1 μL of PrimePCR™ ddPCR™ Expression Probe Assay targeting the nuclear B-Actin (Actb) gene (HEX). Thermal cycling consisted of 95°C for 10 min, followed by 40 cycles of 94°C for 30 s and 55°C for 1 min, and a final step at 98°C for 10 min. A ramp rate of 2°C/s was used for all steps. Droplets were read using a QX200 ddPCR Droplet Reader (Bio-Rad Laboratories), and data were analyzed using QuantaSoft Analysis software, version 1.7.4.0917 (Bio-Rad Laboratories).

Copy numbers were normalized to the initial sample volume analyzed (200 μL).

### Cell damage markers: Caspase-cleaved keratin 18 (CK18) and Adenylate kinase (AK)

Aspecific cell death was evaluated by measuring the release of adenylate kinase (AK) from damaged cells using the ToxiLight™ bioassay kit (Lonza Bioscience, Basel, Switzerland). Bioluminescent signals were then acquired using a Luminometer (Glomax Luminometer, Promega, Madison, WI, USA).

Epithelial cell apoptosis was assessed by measuring the release of caspase-cleaved CK18 with a commercially available immunoassay based on the use of M30 monoclonal antibody that specifically detects the neoepitope ccK18/K18-Asp396 (Cusabio Technology LLC, Houston, USA). Absorbance readings were carried out using a multi-mode microplate reader (Synergy HTX).

## Evaluations performed on rat lung biopsies

### Quantification of mtDNA copy numbers in lung tissue

Total DNA was extracted from 25 mg of frozen lung tissue using the QIAamp DNA Mini Kit (QIAGEN, Hilden, Germany), according to the manufacturer’s instructions. DNA concentration was measured using a NanoDrop ND-1000 spectrophotometer (Thermo Fisher Scientific, Waltham, MA, USA)..

Mitochondrial DNA was quantified by droplet digital PCR (ddPCR). Each reaction had a final volume of 20 μL and contained 20 ng of total DNA, 11 μL of 2× ddPCR Supermix for Probes (Bio-Rad Laboratories, Hercules, CA, USA), 1.1 μL of PrimePCR™ ddPCR™ Expression Probe Assay targeting the mitochondrial NADH dehydrogenase subunit 2 (ND2) gene (FAM), and 1.1 μL of PrimePCR™ ddPCR™ Expression Probe Assay targeting the nuclear B-Actin (Actb) gene (HEX). Thermal cycling consisted of 95°C for 10 min, followed by 40 cycles of 94°C for 30 s and 55°C for 1 min, and a final step at 98°C for 10 min. A ramp rate of 2°C/s was used for all steps. Droplets were read using a QX200 ddPCR Droplet Reader (Bio-Rad Laboratories), and data were analyzed using QuantaSoft Analysis software, version 1.7.4.0917 (Bio-Rad Laboratories).

Tissue mtDNA abundance was normalized to the nuclear Actb target and expressed as the ND2-to-Actb copy-number ratio.

### ATP content assessment

Snap frozen lung samples were homogenized in trichloroacetic acid (Sigma-Aldrich) at a concentration of 50 mg/ml. Lung homogenates were subjected to centrifugation at 20817 g for 15 min at 4°C (Heraeus Multifuge X3R) and then supernatants were collected and diluted 1:30 using 0.1 M Tris-acetate, pH 7.75 (Millipore, Merck KGaA). Next, 10 µl of each sample were dispensed in a blank 96-well plate and 90 µL of luciferase (Enliten ATP Assay System, Promega) were automatically added during the analysis in the Luminometer (Glomax Luminometer, Promega). Bioluminescent signals were immediately detected. ATP concentration was calculated by means of a standard curve that ranged from 10^-4^ M to 10^-10^ M (rATP 10 mM, Promega).

### ATP derivatives by High-performance liquid chromatography (HPLC)

Snap frozen tissue biopsies (40-50 mg) were homogenized in 2.5% trichloroacetic acid (Sigma-Aldrich) at a concentration of 50 mg/ml. Lung homogenates were subjected to centrifugation at 20817 g for 15 min at 4°C (Heraeus Multifuge X3R) and then supernatants were analyzed in duplicates by High-performance liquid chromatography (HPLC) with a mass spectrometer as detector (UltiMate 3000, Thermo Fisher Scientific) using a reversed phase C18 HPLC column (Zorbax RRHD SB-C18, 5micron, 4.6x250 mm, Agilent, Santa Clara, CA, US), with a mobile phase containing 0.1% formic acid in water (fase A) and CH3CN (fase B). The signal was detected by UV absorbance at 254 nm. Energy charge defined as: (ATP + 0.5 x ADP) / (ATP + ADP + AMP) was calculated as previously described (11).

### RNA purification and analysis of the expression of selected genes

Snap frozen tissue biopsies (40-50 mg) were homogenized on ice with an Ultra-Turrax tissue homogenizer (IKA Labortechnik, Staufen, Germany) in an appropriate volume of a guanidine isothiocyanate lysis buffer (Qiagen) supplemented with 14.3 M β-mercaptoethanol (Sigma-Aldrich). Tissue lysates were added v/v to 70 % ethanol and immediately transferred to RNeasy MinElute columns (Qiagen). The RNA bound to RNeasy column was subjected to a DNase I (Qiagen) treatment. Eluated RNA was quantified by absorbance measurement with a Nanodrop ND-100 spectrophotometer (Nanodrop Technologies, Wilmington, DE). Each sample showed a 260/280 ratio between 1.8 and 2.0.

Reverse transcription of RNA samples was performed using the RT² First Strand Kit (Qiagen), starting from 0.5–1 µg of total RNA per reaction, following the manufacturer’s protocol.

Expression of selected genes was evaluated by means of real-time PCR using RT^2^ Profiler PCR Arrays (Qiagen). Briefly, cDNA samples were loaded onto the plates (Qiagen), containing pre-dispensed gene-specific primer sets targeting genes of interest (GOI) (Supplementary Table 1), along with 5 reference genes. Real-time PCR was performed using a QuantStudio Real-Time PCR system (Applied Biosystems, Thermo Fisher Scientific Inc.) with the following cycling conditions: 95°C for 10 min, followed by 40 cycles of 95°C for 15 s and 60°C for 1 min.

Relative quantification was obtained with the comparative Ct method (ΔCt), using the average Ct across samples from the native group as calibrator. Then, mRNA relative quantities were normalized to the geometric mean of selected reference genes (Rplp1, Sdha, B2m, Actb). Gene expression of lungs from the native group was used as baseline reference.

# Supplementary tables

## Supplementary Table 1. List of genes assessed by real-time PCR

| Gene of interest | | | | | Reference genes |
| --- | --- | --- | --- | --- | --- |
| Aifm1 | Ccl7 | Hmox1 | Nos2 | Tlr4 | Actb |
| Akt1 | Ccr1 | Hsf1 | Nox1 | Tnf | B2m |
| Apaf1 | Cd40 | Ifna1 | Nox4 | Tp53 | Hprt1 |
| App | Cidea | Il10 | Nrg1 | Ucp1 | Rplp1 |
| Atg7 | Cideb | Il17a | Parp1 | Ucp2 | Sdha |
| Atp5f1b | Cidec | Il18 | Pdcd1 | Ucp3 |  |
| Bad | Cox15 | Il1b | Pdcd2 | Uqcrc1 |  |
| Bag1 | Cx3cl1 | Il6 | Pdcd4 | Xiap |  |
| Bcl2 | Cxcl12 | Ifng | Pparg |  |  |
| Bcl2l1 | Cxcr2 | Mapk14 | Ppif |  |  |
| Bcl2l11 | Cybb | Mapk9 | Prdx2 |  |  |
| Bdnf | Diablo | Mdh2 | Prdx4 |  |  |
| Bex3 | Edn1 | Mfn1 | Prkcd |  |  |
| Birc2 | Fas | Mmp9 | Pten |  |  |
| Bmp2 | Fos | Myc | Rela |  |  |
| Casp3 | Foxo3 | Ndufa1 | Serpinh1 |  |  |
| Casp8 | Gpx1 | Nfe2l2 | Sirt1 |  |  |
| Casp9 | Gulp1 | Nfkb1 | Sod2 |  |  |
| Cat | Hif1a | Nlrp1a | Tgfb1 |  |  |
| Ccl2 | Hmgb1 | Nol3 | Tlr3 |  |  |

## Supplementary Table 2. Hemodynamics and airway parameter monitoring during EVLP

| **Group** | **Time** | **mPAP (mmHg)** | **PVR (mmHg*min/ml)** | **Ppeak (cmH2O)** |
| --- | --- | --- | --- | --- |
| **EVLP** |  |  |  |  |
|  | **0** | 4.2±0.4 | 2.8±0.3 |  |
|  | **5** | 4.2±0.5 | 2.8±0.3 |  |
|  | **10** | 4±0.5 | 2.1±0.2 |  |
|  | **15** | 4±0.7 | 1.5±0.2 |  |
|  | **20** | 4.6±0.8 | 1.2±0.1 |  |
|  | **25** | 4.4±0.7 | 0.8±0.1 | 14.7±0.5 |
|  | **30** | 5±0.6 | 0.8±0 | 16.6±0.6 |
|  | **35** | 4.8±0.5 | 0.6±0 | 16.5±0.7 |
|  | **40** | 4.8±0.5 | 0.6±0 | 16.7±0.4 |
|  | **45** | 5.6±0.2 | 0.7±0 | 15.7±0.5 |
|  | **60** | 5.4±0.4 | 0.7±0 | 16.6±0.6 |
|  | **120** | 5.8±0.5 | 0.8±0.1 | 17.2±0.3 |
|  | **180** | 5.6±0.5 | 0.7±0.1 | 17±0.5 |
| **EVLP+T3** |  |  |  |  |
|  | **0** | 3.6±0.8 | 2.6±0.4 |  |
|  | **5** | 2.7±0.3 | 2±0.1 |  |
|  | **10** | 2.4±0.3 | 1.4±0.1 |  |
|  | **15** | 2.6±0.2 | 1.1±0 |  |
|  | **20** | 2.9±0.1 | 0.9±0 |  |
|  | **25** | 3.6±0.5 | 0.7±0.1 | 14.9±0.9 |
|  | **30** | 3.8±0.3 | 0.6±0 | 15.5±0.6 |
|  | **35** | 3.9±0.3 | 0.6±0 | 16.2±0.7 |
|  | **40** | 4.1±0.3 | 0.6±0 | 16.3±0.4 |
|  | **45** | 4.2±0.1 | 0.6±0 | 14.3±1.4 |
|  | **60** | 4.8±0.4 | 0.7±0 | 17±0.9 |
|  | **120** | 5.1±0.4 | 0.7±0 | 16.6±0.4 |
|  | **180** | 5.5±0.4 | 0.8±0 | 16.6±1.6 |
| **Ischemia+EVLP** | |  |  |  |
|  | **0** | 4.8±0.2 | 3.2±0.2 |  |
|  | **5** | 4.3±0.4 | 2.9±0.2 |  |
|  | **10** | 4.3±0.4 | 2.3±0.2 |  |
|  | **15** | 4.8±0.4 | 1.6±0.2 |  |
|  | **20** | 5.1±0.4 | 1.2±0.1 |  |
|  | **25** | 5.5±0.5 | 1±0.1 | 16±1 |
|  | **30** | 6±0.4 | 0.9±0 | 15.7±0.9 |
|  | **35** | 5.8±0.4 | 0.8±0 | 15.6±1.5 |
|  | **40** | 5.8±0.4 | 0.7±0 | 17.6±0.5 |
|  | **45** | 6±0.4 | 0.8±0 | 17.8±0.2 |
|  | **60** | 6.2±0.5 | 0.8±0 | 17.3±0.6 |
|  | **120** | 7±0.4 | 0.9±0 | 17.1±0.7 |
|  | **180** | 7±0.5 | 0.9±0 | 17.3±0.7 |
| **Ischemia+EVLP+T3** | | |  |  |
|  | **0** | 5.2±1 | 3.3±0.3 |  |
|  | **5** | 4.4±0.4 | 2.8±0.1 |  |
|  | **10** | 4.2±0.4 | 2.2±0 |  |
|  | **15** | 4.6±0.3 | 1.7±0 |  |
|  | **20** | 5±0.4 | 1.3±0 |  |
|  | **25** | 5.4±0.4 | 1±0 | 15.9±0.8 |
|  | **30** | 5.4±0.3 | 0.8±0 | 17±0.5 |
|  | **35** | 5.4±0.4 | 0.7±0 | 16.7±0.5 |
|  | **40** | 5.5±0.3 | 0.7±0 | 16.5±0.4 |
|  | **45** | 5.7±0.3 | 0.7±0 | 14.6±1.3 |
|  | **60** | 5.8±0.3 | 0.7±0 | 17±0.6 |
|  | **120** | 6.1±0.3 | 0.8±0 | 16.9±0.4 |
|  | **180** | 6.8±0.3 | 0.8±0 | 16.3±0.8 |

## Supplementary Table 3. Perfusate gas-analysis throughout the EVLP procedure

Two-way Repeated measures ANOVA; Tukey’s post hoc test

WO, wash-out

|  |  | **Experimental group** | | | |  |
| --- | --- | --- | --- | --- | --- | --- |
|  |  | **EVLP** | | **Ischemia+EVLP** | |  |
| **Variable** | **Time** | **-** | **T3** | **-** | **T3** | **p-value** |
| **K+ (mmol/L)** | |  |  |  |  | **<0.001int** |
|  | **WO** | 4.66±0.05 | 4.72±0.02 | 4.87±0.03 | 4.92±0.02 |  |
|  | **1 h** | 4.82±0.02 | 4.78±0.02 | 4.9±0 | 4.83±0.02 |  |
|  | **3 h** | 5.13±0.04 | 5.03±0.03 | 5.14±0.02 | 5.14±0.03 |  |
| **Na+ (mmol/L)** | |  |  |  |  | **<0.001** |
|  | **WO** | 154.2±0.97 | 151.8±0.97 | 155.3±0.33 | 152.4±0.93 |  |
|  | **1 h** | 155.2±0.53 | 154.4±0.92 | 157.6±0.27 | 155.1±0.57 |  |
|  | **3 h** | 162.2±0.71 | 159±1.23 | 163.8±0.39 | 162.2±1.1 |  |
| **Ca++ (mmol/L)** | |  |  |  |  | **<0.001** |
|  | **WO** | 0.93±0.01 | 0.93±0.01 | 0.95±0 | 0.94±0.01 |  |
|  | **1 h** | 0.91±0.01 | 0.85±0.01 | 0.93±0.01 | 0.85±0.02 |  |
|  | **3 h** | 0.99±0.01 | 0.84±0.01 | 0.96±0.02 | 0.86±0.01 |  |
| **Cl- (mmol/L)** | |  |  |  |  | 0.262 |
|  | **WO** | 122.4±0.98 | 121.8±1.88 | 123.3±0.67 | 122±1.38 |  |
|  | **1 h** | 125.6±0.34 | 127.2±2.96 | 125.6±0.45 | 123±1 |  |
|  | **3 h** | 128.8±0.66 | 131.3±1.36 | 130.8±0.53 | 128.1±0.85 |  |
| **pH** |  |  |  |  |  | **0.002** |
|  | **WO** | 7.1±0.01 | 7.11±0.01 | 7.08±0.01 | 7.09±0.01 |  |
|  | **1 h** | 7.23±0.03 | 7.28±0.03 | 7.18±0.02 | 7.31±0.02 |  |
|  | **3 h** | 7.26±0.02 | 7.3±0.03 | 7.2±0.02 | 7.33±0.02 |  |
| **HCO3- (mmol/L)** | | |  |  |  | **<0.001** |
|  | **WO** | 10.82±0.17 | 11.14±0.16 | 10.5±0.17 | 10.88±0.2 |  |
|  | **1 h** | 10.39±0.23 | 11.65±0.17 | 10.71±0.15 | 11.31±0.11 |  |
|  | **3 h** | 10.32±0.17 | 11.65±0.28 | 10.21±0.22 | 11.06±0.21 |  |
| **cBase(Ecf) (mmol/L)** | | |  |  |  | **0.019int** |
|  | **WO** | -16.9±0.31 | -16.5±0.07 | -17.5±0.13 | -17±0.19 |  |
|  | **1 h** | -15.7±0.19 | -13.8±0.34 | -16±0.22 | -13.8±0.35 |  |
|  | **3 h** | -15.5±0.18 | -13.6±0.43 | -16.4±0.15 | -13.8±0.34 |  |

## Supplementary Table 4. Perfusate glucose and lactate concentration over EVLP

Two-way Repeated measures ANOVA; Tukey’s post hoc test

WO, wash-out

|  |  | **Experimental group** | | | |  |
| --- | --- | --- | --- | --- | --- | --- |
|  |  | **EVLP** | | **Ischemia+EVLP** | |  |
| **Variable** | **Time** | **-** | **T3** | **-** | **T3** | **p-value** |
| **Glucose (mg/dL)** | |  |  |  |  | 0.840 |
|  | **1 h** | 190±2.42 | 200.5±12.99 | 184±2.28 | 222.75±33.9 |  |
|  | **3 h** | 190.2±2.22 | 189.16±16.17 | 189.6±3.55 | 179.8±18.92 |  |
| **Lactate (mmol/L)** | |  |  |  |  | **0.003** |
|  | **1 h** | 0.5±0.13 | 0.1±0.02 | 0.22±0.04 | 0.88±0.48 |  |
|  | **3 h** | 1.04±0.16 | 0.51±0.06 | 0.7±0.07 | 1.1±0.33 |  |

## Supplementary Table 5. Expression values of selected genes in lung homogenates

Data are expressed as mean±SEM of normalized quantities calculated using the ΔCt method.

One-way ANOVA, Tukey’s post hoc test.

Two-way ANOVA restricted to perfused lungs, considering Ischemia and T3 treatment as testing factors; Tukey’s post hoc test. * denotes p-value for effect of T3 treament. int denotes the presence of an interactive effect between Ischemia and T3 treatment.

|  |  | **Experimental group** | | | | | | **p-value** | |
| --- | --- | --- | --- | --- | --- | --- | --- | --- | --- |
|  |  |  |  | **EVLP** | | **Ischemia+EVLP** | |  |  |
| **Pattern** | **Gene** | **Native** | **Ischemia** | **-** | **T3** | **-** | **T3** | **1-way** | **2-way *** |
| **Cell death** | | | | | | | | |  |
|  | **Fas** | 1.09±0.1 | 1.44±0.15 | 2.26±0.35 | 3.78±0.6 | 3.03±0.12 | 2.71±0.35 | 0.002 | 0.002 |
|  | **Casp3** | 1.11±0.04 | 1.49±0.08 | 1.94±0.22 | 2.39±0.33 | 2.45±0.14 | 1.9±0.1 | 0.002 |  |
|  | **Cd40** | 1.14±0.12 | 1.52±0.18 | 3.98±0.77 | 3.95±0.37 | 3.42±0.87 | 3.27±0.34 | 0.004 |  |
|  | **Casp8** | 1.15±0.08 | 1.82±0.17 | 2.49±0.43 | 3.49±0.82 | 2.19±0.19 | 2.06±0.07 | 0.012 | 0.042 |
| **Cell death - inhibitors** | | | | | | | | |  |
|  | **Birc2** | 1.16±0.13 | 1.52±0.15 | 2.87±0.75 | 3.03±0.22 | 2.34±0.2 | 2.62±0.18 | 0.010 | 0.025 |
|  | **Bag1** | 1.13±0.07 | 1.54±0.16 | 1.61±0.31 | 1.98±0.17 | 1.61±0.09 | 1.71±0.04 |  | 0.023 |
|  | **Nol3** | 1.1±0.09 | 2.58±0.29 | 2.21±0.23 | 2.51±0.47 | 1.67±0.32 | 1.95±0.32 |  |  |
|  | **Xiap** | 1.12±0.06 | 1.26±0.1 | 1.19±0.22 | 1.11±0.08 | 1.15±0.1 | 1.1±0.1 |  |  |
| **Cell death - mitochondrial pathway** | | | | | | | | |  |
|  | **Bcl2l11** | 1.16±0.11 | 2.03±0.3 | 4.11±0.47 | 5.06±0.74 | 2.82±0.27 | 4.34±0.23 | 0.001 | <0.001 |
|  | **Ppif** | 1.14±0.15 | 1.98±0.1 | 3.07±0.74 | 3.66±0.24 | 2.3±0.25 | 2.49±0.24 | 0.004 | 0.017 |
|  | **Bcl2** | 1.15±0.11 | 2.23±0.21 | 1.69±0.36 | 2.8±0.19 | 1.4±0.1 | 1.72±0.19 | 0.005 | 0.009 |
|  | **Aifm1** | 1.13±0.1 | 1±0.23 | 1.04±0.4 | 0.54±0.13 | 1.86±0.17 | 1.77±0.3 | 0.009 |  |
|  | **Casp9** | 1.07±0.07 | 2.28±0.25 | 1.75±0.31 | 2.54±0.34 | 1.16±0.12 | 1.37±0.13 | 0.010 | 0.008int |
|  | **Tp53** | 1.16±0.08 | 4.72±1.15 | 4.81±0.32 | 6.02±1.04 | 2.87±0.84 | 3.75±0.36 | 0.011 |  |
|  | **Apaf1** | 1.12±0.06 | 1.99±0.15 | 2.13±0.46 | 3.21±0.31 | 2.21±0.15 | 2.35±0.12 | 0.011 | <0.001 |
|  | **Bad** | 1.12±0.12 | 2.14±0.12 | 1.8±0.37 | 2.66±0.42 | 1.1±0.25 | 1.52±0.32 | 0.015 | 0.032int |
|  | **Foxo3** | 1.1±0.1 | 2.68±0.25 | 2.9±0.31 | 2.84±0.87 | 2.3±0.16 | 1.68±0.17 | 0.025 |  |
|  | **Bcl2l1** | 1.09±0.04 | 2.09±0.2 | 1.27±0.27 | 1.07±0.11 | 1.19±0.03 | 1.32±0.11 | 0.027 |  |
|  | **Diablo** | 1.11±0.05 | 2.21±0.27 | 1.47±0.28 | 2.8±0.78 | 1.46±0.38 | 0.99±0.34 |  | 0.006int |
| **Growth and differentiation** | | | | | | | | |  |
|  | **Nrg1** | 1.15±0.09 | 1.85±0.25 | 5.01±0.47 | 5.18±0.44 | 7.15±1.12 | 7.4±1.73 | 0.002 | 0.023 |
|  | **Bmp2** | 1.08±0.21 | 3.24±0.16 | 3.45±0.97 | 4.18±0.59 | 3.99±0.25 | 2.98±0.28 | 0.010 | 0.023 |
|  | **App** | 1.12±0.08 | 1.76±0.21 | 1.3±0.31 | 1.94±0.28 | 1.46±0.03 | 1.67±0.03 |  | 0.045 |
|  | **Bdnf** | 1.13±0.12 | 1.21±0.17 | 1.7±0.42 | 2.01±0.31 | 2.1±0.32 | 2.61±0.36 |  | 0.011 |
|  | **Bex3** | 1.16±0.09 | 1.31±0.21 | 0.82±0.18 | 1.03±0.13 | 0.96±0.06 | 1.23±0.1 |  |  |
|  | **Edn1** | 1.23±0.22 | 2.94±0.8 | 1.1±0.18 | 1.3±0.17 | 0.83±0.21 | 1.37±0.55 |  |  |
|  | **Pparg** | 1.16±0.23 | 1.11±0.31 | 1.15±0.47 | 0.64±0.05 | 1.49±0.1 | 1.68±0.2 |  |  |
| **Inflammation and innate immunity** | | | | | | | | |  |
|  | **Rela** | 1.11±0.09 | 2.71±0.17 | 4.13±0.43 | 7.39±2.86 | 3.05±0.17 | 3.3±0.17 | 0.001 | 0.042 |
|  | **Il1b** | 1.07±0.12 | 1.86±0.36 | 15.11±3.29 | 24.64±4.71 | 17.34±4.34 | 15.45±1.01 | 0.002 | 0.005 |
|  | **Il6** | 1.21±0.07 | 2.02±0.37 | 435.75±159.99 | 218.23±91.54 | 642.01±105.43 | 661.72±550.86 | 0.002 |  |
|  | **Tnf** | 0.91±0.18 | 2.77±0.89 | 29.16±5.79 | 33.55±6.35 | 14.32±5.57 | 19.38±1.84 | 0.002 | 0.029 |
|  | **Ccl2** | 1.25±0.15 | 4.81±2.16 | 29.86±9.32 | 20.52±6.87 | 80.57±21.6 | 89.03±61.5 | 0.002 |  |
|  | **Nlrp1a** | 1.14±0.13 | 2.27±0.25 | 1.8±0.48 | 44.82±42.69 | 0.5±0.06 | 1.04±0.22 | 0.002 |  |
|  | **Ccl7** | 1.21±0.19 | 2.38±0.5 | 16.89±3.74 | 16.34±4.38 | 26.85±6.53 | 47.33±29.27 | 0.002 |  |
|  | **Tgfb1** | 1.09±0.05 | 3.42±0.74 | 4.28±0.89 | 4.7±0.57 | 1.86±0.24 | 2.65±0.15 | 0.004 |  |
|  | **Il10** | 1.68±0.42 | 3.08±1.28 | 7.55±1 | 14.19±2.32 | 12.64±4.37 | 14.15±4.05 | 0.005 | 0.004 |
|  | **Nfkb1** | 1.1±0.05 | 3.37±0.53 | 4.68±1.04 | 6.07±0.54 | 3.08±1 | 3.1±0.17 | 0.005 | 0.042 |
|  | **Cx3cl1** | 1.25±0.2 | 4.64±0.61 | 10.57±2.64 | 14.98±4.14 | 6.51±3.93 | 6.57±1.28 | 0.007 | 0.047int |
|  | **Cxcl12** | 1.19±0.14 | 1.96±0.22 | 1.58±0.22 | 2.01±0.22 | 1.07±0.12 | 1.31±0.17 | 0.021 |  |
|  | **Mmp9** | 1.2±0.23 | 1.97±0.61 | 9.96±5.79 | 48.44±44.97 | 0.88±0.39 | 1±0.12 | 0.022 |  |
|  | **Tlr4** | 1.16±0.02 | 1.11±0.12 | 1.98±0.36 | 1.38±0.05 | 1.45±0.11 | 1.67±0.16 | 0.028 |  |
|  | **Ccr1** | 1.11±0.06 | 1.51±0.16 | 1.3±0.26 | 1.19±0.2 | 1.37±0.2 | 1.37±0.36 |  |  |
|  | **Cxcr2** | 1.11±0.18 | 0.93±0.13 | 0.4±0.08 | 1.03±0.29 | 0.82±0.14 | 0.78±0.09 |  |  |
|  | **Hmgb1** | 1.09±0.14 | 1.13±0.2 | 0.52±0.13 | 0.71±0.03 | 0.86±0.11 | 0.86±0.24 |  |  |
|  | **Ifna1** | 1.48±0.27 | 1.67±0.29 | 1.02±0.34 | 1.81±0.43 | 1.05±0.25 | 0.69±0.07 |  | 0.030int |
|  | **Ifng** | 2.12±0.98 | 1.97±0.44 | 1.59±0.66 | 1.74±0.19 | 0.84±0.45 | 6.07±4.9 |  |  |
|  | **Il17a** | 1.24±0.62 | 2.04±0.18 | 2.64±0.5 | 0.94±0.81 | 0.65±0.5 | 0.55±0.5 |  |  |
|  | **Il18** | 1.19±0.21 | 1±0.2 | 1.24±0.29 | 0.96±0.12 | 1.35±0.35 | 1.2±0.44 |  |  |
|  | **Tlr3** | 1.17±0.13 | 1.54±0.59 | 0.91±0.07 | 0.87±0.03 | 0.86±0.08 | 1.11±0.12 |  |  |
| **Mitochondrial energy metabolism** | | | | | | | | |  |
|  | **Cox15** | 1.08±0.05 | 2.49±0.31 | 2±0.42 | 3.12±0.49 | 1.19±0.12 | 1.85±0.26 | 0.008 | 0.041 |
|  | **Atp5f1b** | 1.12±0.07 | 1.1±0.28 | 0.91±0.14 | 0.8±0.07 | 1.1±0.02 | 1.21±0.15 |  |  |
|  | **Mdh2** | 1.12±0.09 | 0.96±0.2 | 0.39±0.11 | 1.15±0.18 | 0.95±0.03 | 1.1±0.14 |  |  |
|  | **Mfn1** | 1.08±0.1 | 1.98±0.28 | 2.83±0.8 | 2.58±0.7 | 2.05±0.15 | 2.66±0.14 |  |  |
|  | **Ndufa1** | 1.13±0.09 | 0.78±0.3 | 0.85±0.08 | 0.66±0.04 | 0.75±0.2 | 1.05±0.11 |  |  |
|  | **Ucp1** | 1.44±0.57 | 3.6±0.72 | 2.09±0.45 | 23.12±20.17 | 2.43±0.24 | 1.25±0.13 |  |  |
|  | **Ucp2** | 1.07±0.09 | 2.02±0.23 | 1.13±0.03 | 1.13±0.08 | 0.84±0.11 | 0.96±0.1 |  |  |
|  | **Ucp3** | 1.57±0.76 | 1.07±0.14 | 1.56±0.08 | 14.75±13.17 | 1.48±0.25 | 1.71±0.33 |  |  |
|  | **Uqcrc1** | 1.2±0.18 | 1.45±0.19 | 1.49±0.14 | 1.6±0.16 | 1.01±0.02 | 1.24±0.1 |  |  |
| **Oxidative stress and antioxidant respoe** | | | | | | | | |  |
|  | **Nos2** | 0.5±0.04 | 1.05±0.16 | 1.8±0.16 | 4.03±1.26 | 3.66±1.42 | 2.37±0.37 | 0.002 |  |
|  | **Sod2** | 1.16±0.07 | 1.33±0.13 | 7.08±2.35 | 4.64±1.56 | 5.45±0.73 | 6.01±1.51 | 0.003 |  |
|  | **Hmox1** | 1.24±0.28 | 2.15±0.19 | 13.44±5.83 | 11.49±2.77 | 12.16±0.92 | 12.19±4.85 | 0.003 |  |
|  | **Nfe2l2** | 1.12±0.03 | 2.55±0.36 | 7.6±1.95 | 5.18±0.98 | 5.34±0.5 | 4.91±0.29 | 0.005 |  |
|  | **Gpx1** | 1.15±0.1 | 1.1±0.22 | 0.5±0.13 | 0.64±0.05 | 0.95±0.05 | 0.94±0.15 | 0.008 |  |
|  | **Cat** | 1.15±0.04 | 1.21±0.31 | 1.21±0.29 | 1.58±0.14 | 1.05±0.18 | 1.21±0.13 |  |  |
|  | **Cybb** | 1.09±0.17 | 1.9±0.21 | 2.1±0.49 | 2.45±0.23 | 1.67±0.23 | 1.72±0.14 |  |  |
|  | **Nox1** | 1.05±0.21 | 1.97±0.2 | 5.79±3.22 | 1.59±0.35 | 1.79±0.27 | 3±1.19 |  |  |
|  | **Nox4** | 1.16±0.13 | 1.7±0.12 | 4.19±2.2 | 1.66±0.19 | 0.8±0.25 | 1.37±0.06 |  |  |
|  | **Prdx2** | 1.18±0.11 | 1.21±0.13 | 1.01±0.18 | 1.32±0.09 | 0.93±0.01 | 0.99±0.08 |  |  |
|  | **Prdx4** | 1.17±0.1 | 1.44±0.22 | 1.19±0.17 | 0.97±0.08 | 1.22±0.06 | 1.18±0.13 |  |  |
| **Stress response and survival** | | | | | | | | |  |
|  | **Mapk14** | 1.08±0.05 | 2.87±0.24 | 1.68±0.23 | 2.46±0.19 | 1.5±0.17 | 2.02±0.1 | 0.001 | 0.015int |
|  | **Sirt1** | 1.13±0.15 | 1.69±0.2 | 5.17±1.85 | 2.39±0.18 | 3.08±0.46 | 3.48±0.32 | 0.003 |  |
|  | **Serpinh1** | 1.17±0.1 | 2.92±0.39 | 1.42±0.16 | 2.07±0.18 | 1.23±0.16 | 1.58±0.2 | 0.003 | 0.036int |
|  | **Akt1** | 1.12±0.07 | 2.2±0.58 | 2.17±0.15 | 3.4±0.25 | 1.61±0.12 | 1.65±0.17 | 0.004 | 0.026 |
|  | **Myc** | 1.13±0.11 | 5.44±1.14 | 12.56±2.26 | 14.9±3.98 | 14.68±5.81 | 6.9±0.87 | 0.007 |  |
|  | **Fos** | 1.48±0.65 | 8.7±1.32 | 2.22±0.15 | 2.4±0.44 | 3.1±0.31 | 2.04±0.77 | 0.008 | 0.025int |
|  | **Hif1a** | 0.9±0.06 | 1.25±0.08 | 1.31±0.34 | 1.21±0.24 | 1.56±0.07 | 2.18±0.23 | 0.012 | 0.02 |
|  | **Parp1** | 1.16±0.15 | 2.74±0.3 | 2.28±0.23 | 3.73±0.4 | 1.62±0.17 | 2.06±0.23 | 0.017 | 0.033 |
|  | **Hsf1** | 1.14±0.19 | 1.76±0.14 | 2.46±0.31 | 2.2±0.25 | 1.34±0.22 | 1.56±0.18 |  |  |
|  | **Mapk9** | 1.1±0.07 | 1.74±0.16 | 2.71±1.09 | 3.08±0.97 | 1.56±0.24 | 1.68±0.15 |  |  |
|  | **Prkcd** | 1.14±0.04 | 1.36±0.09 | 1.04±0.14 | 1.03±0.2 | 0.96±0.06 | 1.13±0.09 |  |  |
|  | **Pten** | 1.15±0.11 | 1.05±0.03 | 1.31±0.25 | 1.29±0.07 | 1.36±0.1 | 1.63±0.09 |  | 0.037 |
| **Other** | | | | | | | | |  |
|  | **Atg7** | 1.05±0.04 | 3.43±0.49 | 2.02±0.59 | 3.51±0.58 | 1.43±0.14 | 1.7±0.03 | 0.002 | 0.005int |
|  | **Cidec** | 1.11±0.13 | 3.26±0.51 | 2.95±0.52 | 6.37±0.95 | 1.96±0.39 | 2.75±0.65 | 0.004 | 0.001 |
|  | **Cideb** | 1.35±0.28 | 3.78±0.63 | 2.94±0.13 | 23.14±20.82 | 1.09±0.16 | 0.9±0.31 | 0.004 |  |
|  | **Cidea** | 1.11±0.1 | 2.32±0.48 | 2.2±0.28 | 2.72±0.32 | 1.78±0.26 | 2.46±0.29 | 0.027 | 0.031 |
|  | **Gulp1** | 1.14±0.1 | 1.04±0.09 | 0.9±0.18 | 1.07±0.04 | 0.91±0.08 | 0.98±0.09 |  |  |
|  | **Pdcd1** | 3.41±1.71 | 9.51±2.13 | 8.43±2.42 | 3.73±2.32 | 5.51±1.28 | 3.54±2.9 |  |  |
|  | **Pdcd2** | 1.15±0.08 | 1.05±0.33 | 1.22±0.2 | 0.91±0.07 | 1.23±0.09 | 1.33±0.16 |  |  |
|  | **Pdcd4** | 1.15±0.08 | 1.95±0.33 | 2.07±0.59 | 2.39±0.22 | 1.53±0.1 | 2.83±1.05 |  | 0.04 |

## Supplementary Table 6. Evaluation of Steen Solution^TM^ hormonal content

|  | **Steen Solution** | | **Steen Solution added with 1 μM T3** |
| --- | --- | --- | --- |
| **Hormone** | **Lot 1** | **Lot 2** | **Lot 1** |
| **Aldosterone** | 4.7 ng/dL | 4.5 ng/dL |  |
| **Estradiol E2** | 83 pmol/L | 87 pmol/L |  |
| **fT3** | 24.2 pmol/L | 19.1 pmol/L | 50 pmol/L |
| **fT4** | 42.1 pmol/L | 37.8 pmol/L |  |
| **Progesterone** | 0.72 nmol/L | 0.69 nmol/L |  |
| **Testosterone** | 3.14 nmol/L | 3.02 nmol/L |  |

## Supplementary Table 7. Effect estimates and 95% CI for selected energetic, apoptotic, and functional outcomes.

Omnibus effect sizes are reported as epsilon-squared (ε²), and pairwise non-parametric effects as Cliff’s delta (δ). Selected outcomes were further examined by exploratory 2 × 2 ANOVA in the four perfused groups. Longitudinal pCO₂ data were analysed using a mixed-effects model. Parametric estimates are mean differences with 95% CIs; Dunn’s and Tukey’s p values are multiplicity-adjusted. Factorial analyses should be interpreted as exploratory because of the limited sample size.

| **Outcome** | **Analysis** | **Effect/comparison** | **Effect estimate (95% CI)** | **p-value** |
| --- | --- | --- | --- | --- |
| AMP/ATP ratio | Kruskal–Wallis | Overall group difference | epsilon^2=0.517 | 0.0073 |
| AMP/ATP ratio | Dunn’s multiple-comparisons test | Ischemia vs EVLP+T3 | Cliff’s delta=1.00 | 0.0458 |
| Bim/Bcl-XL ratio | Kruskal–Wallis test | Overall difference among groups | epsilon^2=0.819 | 0.0002 |
| Bim/Bcl-XL ratio | Dunn’s multiple-comparisons test | Native vs EVLP+T3 | Cliff’s delta=-1.00 | 0.0054 |
| Bim/Bcl-XL ratio | Dunn’s multiple-comparisons test | Ischemia vs EVLP+T3 | Cliff’s delta=-1.00 | 0.0019 |
| Bim/Bcl-XL ratio | Exploratory two-way ANOVA | T3 main effect: Control − T3 | −0.9561 (−1.899 to −0.0130) | 0.0473 |
| Bim/Bcl-XL ratio | Exploratory two-way ANOVA | Ischemia main effect: No ischemia − Ischemia | 1.161 (0.2175 to 2.104) | 0.0192 |
| Bim/Bcl-XL ratio | Exploratory two-way ANOVA | Ischemia × T3 interaction (difference-in-differences) | −0.0962 (−1.982 to 1.790) | 0.9149 |
| Energy charge | Kruskal–Wallis test | Overall difference among groups | epsilon^2=0.510 | 0.0078 |
| FAS expression | Kruskal–Wallis test | Overall difference among groups | epsilon^2=0.576 | 0.0021 |
| FAS expression | Dunn’s multiple-comparisons test | Native vs EVLP+T3 | Cliff’s delta=-1.00 | 0.0267 |
| FAS expression | Dunn’s multiple-comparisons test | Native vs Ischemia+EVLP+T3 | Cliff’s delta=-1.00 | 0.0143 |
| FAS expression | Exploratory two-way ANOVA | T3 main effect: Control − T3 | −0.004092 (−0.006722 to −0.001463) | 0.0045 |
| FAS expression | Exploratory two-way ANOVA | Ischemia main effect: No ischemia − Ischemia | 0.00002551 (−0.002604 to 0.002655) | 0.9838 |
| FAS expression | Exploratory two-way ANOVA | Ischemia × T3 interaction (difference-in-differences) | 0.002394 (−0.002865 to 0.007652) | 0.3489 |
| FAS expression | Tukey’s multiple-comparisons test | Ischemia+EVLP vs Ischemia+EVLP+T3 | −0.005289 (−0.01031 to −0.000269) | 0.0371 |
| pCO2 | Mixed-effects model, Tukey’s multiple-comparisons test | 60 min: EVLP vs Ischemia+EVLP | −5.414 (−10.76 to −0.0653) | 0.0464 |
| pCO2 | Mixed-effects model, Tukey’s multiple-comparisons test | 60 min: Ischemia+EVLP vs Ischemia+EVLP+T3 | 5.694 (0.3453 to 11.04) | 0.0333 |
| Tissue ATP content | Exploratory two-way ANOVA | T3 main effect: Control − T3 | 98.93 (−101.0 to 298.9) | 0.3065 |
| Tissue ATP content | Exploratory two-way ANOVA | Ischemia main effect: No ischemia − Ischemia | 323.1 (123.1 to 523.0) | 0.0038 |
| Tissue ATP content | Exploratory two-way ANOVA | Ischemia × T3 interaction (difference-in-differences) | 595.4 (195.5 to 995.2) | 0.0065 |
| Tissue ATP content | Tukey’s multiple-comparisons test | EVLP vs EVLP+T3 | 396.6 (35.35 to 757.8) | 0.0295 |
| Tissue ATP content | Tukey’s multiple-comparisons test | Ischemia+EVLP vs Ischemia+EVLP+T3 | −198.8 (−602.6 to 205.1) | 0.5022 |

# Supplementary figures

## Supplementary Figure 1. Experimental design and treatment randomization scheme.

Thirty lungs were allocated to 6 experimental groups.

Allocation to EVLP procedure (n=20) or non-EVLP (n=10) was established a priori for logistical reasons.

Within the EVLP branch, lungs were randomly assigned to undergo WI followed by ISP+CI (n=10) or ISP alone (n=10). A second round of randomization was performed to allocate lungs to receive either T3 (n=5 for each group) or saline (n=5 for each group).

Abbreviations: CI, cold ischemia; EVLP, ex vivo lung perfusion; ISP, in situ perfusion; WI, warm ischemia.

## Supplementary Figure 2. Induction of Thyroid hormone responsive (Thrsp1) gene in lungs treated with T3

Kruskal-Wallis test followed by Dunn’s multicomparison procedure

## Supplementary Figure 3. Lung compliance over the EVLP procedure

Two-way ANOVA, Tukey’s post hoc test

## Supplementary Figure 4. K+ release into the perfusate during EVLP

Two-way ANOVA, Tukey’s post hoc test

## Supplementary Figure 5. ccCK18 release into the perfusate during EVLP

Two-way ANOVA, Tukey’s post hoc test

## Supplementary Figure 6. Association between mtDNA and AK release in the perfusate.

Plot: Each point represents one paired measurement. The solid line represents the linear fit, and the dotted lines indicate its 95% confidence interval.

Statistics: Spearman’s r = 0.538, 95% CI 0.349–0.686, p<0.0001.

## Supplementary Figure 7. Association between mtDNA and ccCK18 release in the perfusate.

Plot: Each point represents one paired measurement. The solid line represents the linear fit, and the dotted lines indicate its 95% confidence interval.

Statistics: Spearman’s r = 0.565, 95% CI 0.380–0.706, p<0.0001.

## Supplementary Figure 8. Association between mtDNA and 8OHdG in the perfusate.

Plot: Each point represents one paired measurement. The solid line represents the linear fit, and the dotted lines indicate its 95% confidence interval.

Statistics: Spearman’s r = 0.0394, 95% CI -0.259–0.331, p=0.793.

## Supplementary Figure 9. Association between tissue mtDNA and tissue ATP content.

Plot: Each point represents one paired measurement. The solid line represents the linear fit, and the dotted lines indicate its 95% confidence interval.

Statistics: Spearman’s r = 0.221, 95% CI -0.552–0.169, p=0.248.

## Supplementary Figure 10. Association between tissue mtDNA and tissue AMP/ATP ratio.

Plot: Each point represents one paired measurement. The solid line represents the linear fit, and the dotted lines indicate its 95% confidence interval.

Statistics: Spearman’s r = 0.0867, 95% CI -0.461–0.314, p=0.667.

# References

1.	Smith AJ, Clutton RE, Lilley E, Hansen KEA, Brattelid T. PREPARE: guidelines for planning animal research and testing. Lab Anim. 2018;52(2):135–41.

2.	Balls M. It’s Time to Reconsider The Principles of Humane Experimental Technique. ATLA Altern to Lab Anim. 2020;48(1):40–6.

3.	Russell WMS, Burch RL. The Principles of Humane Experimental Technique. Methuen Co, Ltd. 1959;

4.	Dondossola D, Lonati C, Battistin M, Vivona L, Zanella A, Maggioni M, et al. Twelve-hour normothermic liver perfusion in a rat model: characterization of the changes in the ex-situ bio-molecular phenotype and metabolism. Sci Rep. 2024;14(1):1–13.

5.	Muller X, Schlegel A, Kron P, Eshmuminov D, Würdinger M, Meierhofer D, et al. Novel Real-time Prediction of Liver Graft Function During Hypothermic Oxygenated Machine Perfusion Before Liver Transplantation. Ann Surg. 2019;

6.	Lonati C, Schlegel A, Battistin M, Merighi R, Carbonaro M, Dongiovanni P, et al. Effluent Molecular Analysis Guides Liver Graft Allocation to Clinical Hypothermic Oxygenated Machine Perfusion. 2021;(Dcd):1–18.

7.	Tiranti V, Viscomi C, Hildebrandt T, Di Meo I, Mineri R, Tiveron C. Loss of ETHE1, a mitochondrial dioxygenase, causes fatal sulfide toxicity in ethylmalonic encephalopathy. Nat Med. 2009;15(2):200–5.

8.	Protti A, Fortunato F, Monti M, Vecchio S, Gatti S, Comi GP, et al. Metformin overdose, but not lactic acidosis per se, inhibits oxygen consumption in pigs. Crit Care. 2012;16(3):1–8.

9.	Monzio Compagnoni G, Kleiner G, Bordoni A, Fortunato F, Ronchi D, Salani S, et al. Mitochondrial dysfunction in fibroblasts of Multiple System Atrophy. Biochim Biophys Acta - Mol Basis Dis [Internet]. 2018;1864(12):3588–97. Available from: https://doi.org/10.1016/j.bbadis.2018.09.018

10.	Bresolin N, Bet L, Moggio M, Meola G, Fortunato F, Comi G, et al. Muscle glucose-6-phosphate dehydrogenase deficiency. J Neurol. 1989;236(4):193–8.

11.	Meszaros AT, Hofmann J, Buch ML, Cardini B, Dunzendorfer-Matt T, Nardin F, et al. Mitochondrial respiration during normothermic liver machine perfusion predicts clinical outcome. EBioMedicine. 2022 Nov;85.
